# HLH‐30/TFEB Rewires the Chaperone Network to Promote Proteostasis Upon Perturbations to the Coenzyme A and Iron–Sulfur Cluster Biosynthesis Pathways

**DOI:** 10.1111/acel.70038

**Published:** 2025-04-30

**Authors:** Rewayd Shalash, Dror Michael Solomon, Mor Levi‐Ferber, Henrik von Chrzanowski, Mohammad Khaled Atrash, Barak Nakar, Matan Yosef Avivi, Hagit Hauschner, Aviya Swisa, Alicia Meléndez, Yaron Shav‐Tal, Sivan Henis‐Korenblit

**Affiliations:** ^1^ The Mina & Everard Goodman Faculty of Life Sciences Bar‐Ilan University Ramat‐Gan Israel; ^2^ Biology Department, Queens College City University of New York (CUNY) New York USA; ^3^ Institute of Nanotechnology and Advanced Materials (BINA) Bar‐Ilan University Ramat‐Gan Israel; ^4^ The Metabolomics Unit at the Kanbar Core Facility, The Mina and Everard Goodman Faculty of Life Sciences Bar‐Ilan University Ramat‐Gan Israel; ^5^ Biology and Biochemistry PhD Programs The Graduate Center of the City University of New York New York USA

**Keywords:** *C. elegans*, chaperones, coenzyme A, HLH‐30, iron–sulfur clusters, pantothenate kinase, protein quality control, proteostasis, TFEB

## Abstract

The maintenance of a properly folded proteome is critical for cellular function and organismal health, and its age‐dependent collapse is associated with a wide range of diseases. Here, we find that despite the central role of Coenzyme A as a molecular cofactor in hundreds of cellular reactions, inhibition of the first and rate‐limiting step in CoA biosynthesis can be beneficial and promote proteostasis. Impairment of the cytosolic iron–sulfur cluster formation pathway, which depends on Coenzyme A, similarly promotes proteostasis and acts in the same pathway. Proteostasis improvement by interference with the Coenzyme A/iron–sulfur cluster biosynthesis pathways is dependent on the conserved HLH‐30/TFEB transcription factor. Strikingly, under these conditions, HLH‐30 promotes proteostasis by potentiating the expression of select chaperone genes, providing a chaperone‐mediated proteostasis shield, rather than by its established role as an autophagy and lysosome biogenesis‐promoting factor. This reflects the versatile nature of this conserved transcription factor, which can transcriptionally activate a wide range of protein quality control mechanisms, including chaperones and stress response genes alongside autophagy and lysosome biogenesis genes. These results highlight TFEB as a key proteostasis‐promoting transcription factor and underscore it and its upstream regulators as potential therapeutic targets in proteostasis‐related diseases.

## Graphical abstract

Proteostasis enhancing treatments are potential therapies for many age‐related diseases. We discovered that interference with the CoA biosynthesis pathway and limiting iron‐sulfur cluster availability improve proteostasis in *C. elegans* . Proteostasis improvement under these conditions depends on the conserved HLH‐30/TFEB transcription factor, which potentiates the expression of chaperone genes. These findings mark CoA biosynthesis and iron‐sulfur cluster availability as upstream regulators of HLH‐30/TFEB and marks HLH‐30/TFEB as a versatile context‐dependent modulator of the entire protein quality control system.

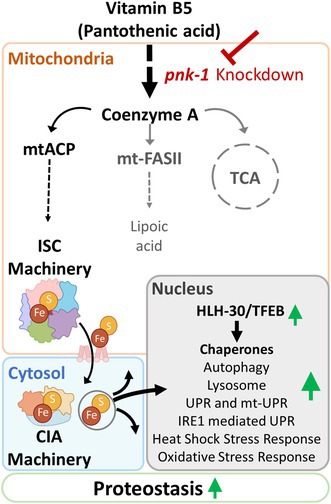

## 1 Introduction

The maintenance of a properly folded proteome is critical for cellular function and organismal health (Labbadia and Morimoto 2015; Meller and Shalgi 2021). High abundance of misfolded proteins drives the formation of insoluble protein aggregates that accumulate within the cell or in its vicinity, where they induce stress responses, jeopardize physiological cellular function, and consequently can lead to the development of proteinopathies, many of which are age‐dependent neurodegenerative diseases affecting a significant proportion of the elderly population (Hoppe and Cohen 2020; Paulson 1999). This underscores the importance of identifying new pathways and mechanisms that promote proteostasis at the cell and organism levels.

To counteract and minimize the damage inflicted by misfolded proteins and maintain proteostasis, a highly conserved protein quality control (PQC) system that repairs or removes damaged proteins has evolved (Jayaraj et al. 2020). Cellular chaperones and their co‐chaperones are central components of the PQC network, and they are implicated in many aspects of protein quality control, including de novo folding of nascent polypeptides, unfolding and reactivation of denatured proteins, and controlled degradation, aggregation, or extrusion of terminally misfolded proteins (Jayaraj et al. 2020; Wentink and Rosenzweig 2023). A series of dedicated stress response pathways allow the PQC system to adapt and expand according to the changing needs of the cell. This flexibility of the PQC is attributed, at least in part, to a set of stress‐related transcription factors that support the expression of genes that reinforce the expression of protein quality control components (Pessa et al. 2024).

Coenzyme A (CoA) is a fundamental conserved cellular cofactor in hundreds of metabolic reactions (Begley et al. 2001). These include the tricarboxylic acid (TCA) cycle, mitochondrial fat metabolism and oxidation, acyl carrier protein (ACP) cofactor activation, sterol and steroid synthesis, protein acetylation, and more (Leonardi et al. 2005; Strauss 2010). *De novo* CoA biosynthesis requires pantothenic acid (PA, also known as vitamin B5), cysteine, and ATP. In this process, pantothenic acid is converted into CoA by a series of five enzymatic reactions (Daugherty et al. 2002). The first step in CoA biosynthesis, which is also the rate‐limiting step, is the phosphorylation of pantothenic acid by pantothenate kinase (PanK) (Robishaw and Neely 1985). In addition, a salvage pathway exists in which the CoA breakdown product pantetheine/4′‐phosphopantetheine can serve as advanced precursors for CoA synthesis by the last two enzymes of the canonical CoA biosynthetic pathway (Srinivasan et al. 2015; Sibon and Strauss 2016).

Consistent with the role of CoA as a central cofactor, mutations in its biosynthetic pathway are associated with various diseases. For example, mutations in the human mitochondrial PanK isoform PANK2 gene cause classical pantothenate kinase‐associated neurodegeneration syndrome (PKAN)—a monogenic neurodegenerative motor‐disorder characterized by movement abnormalities, vision impairment, dementia, iron accumulation in the brain, ventricular dysfunction, and ultimately premature death (Gregory and Hayflick 1993; Zhou et al. 2001; Audam et al. 2021). Similarly, mutations in the COASY gene, which acts downstream in the CoA biosynthesis pathway, result in a related disorder known as CoA synthase protein‐associated neurodegeneration (CoPAN) (Di Meo et al. 2020; Dusi et al. 2014).

Among its many roles, CoA is required for the biogenesis of Iron–sulfur (Fe/S) clusters (ISCs) (Lill and Freibert 2020). ISCs are iron and sulfur ions assembled into scaffold complexes in the form of 2Fe–2S and 4Fe–4S clusters (Shi et al. 2021). Fe‐S clusters can accept or donate electrons to carry out oxidation/reduction reactions and facilitate electron transport (Rouault 2015). In the nucleus, Fe‐S clusters are cofactors in several crucial DNA repair enzymes. All in all, ISCs are cofactors of more than 200 Fe/S proteins and are implicated in a variety of metabolic reactions (Paul and Lill 2015). ISCs are usually assembled in the mitochondria by dedicated ISC machinery (Paul and Lill 2015). Whereas ISCs assemble with a variety of protein complexes in the mitochondria, ISCs generated in the mitochondria can also be exported to the cytoplasm and nucleus via dedicated transporters such as the ABC transporter ABCB7, which deliver them to the cytosolic iron–sulfur protein assembly (CIA) machinery (Lill and Mühlenhoff 2008). In the cytoplasm, specialized proteins such as CIAO1 and CIA2B load the ISCs onto target cytosolic Fe–S proteins (Lill et al. 2014). Deregulation of mitochondrial and cytoplasmic ISC components and substrates have been linked to numerous human diseases (Sheftel et al. 2010; Maio and Rouault 2020).

In this work, we explored how deficiencies in CoA and ISC biosynthetic pathways affect the proteostasis network. Unexpectedly, we find that mild interference with the CoA and cytosolic ISCs biosynthetic pathways improve the resistance of the animals to a wide variety of proteostasis challenges. These conditions are associated with activation of the HLH‐30/TFEB transcription factor, which is mostly known as a central autophagy and lysosome biogenesis‐promoting factor. Strikingly, we discovered that under these conditions, HLH‐30 promoted proteostasis by potentiating the expression of chaperone genes. These findings mark CoA and ISC availability as potential upstream regulators of the HLH‐30/TFEB transcription factor and highlight this conserved transcription factor as a versatile context‐dependent modulator of the PQC system.

## 2 Results

### 2.1 *pnk‐1* RNAi Limits Foci Formation in a PolyQ Disease Model

PANK deficiency is associated with neurodegenerative diseases in humans, and knockout of its homologs results in unhealthy, short‐lived animals in a variety of model organisms including *C. elegans* (Wu et al. 2009; Samuelson et al. 2007; Srinivasan et al. 2015; Mignani et al. 2021). Nevertheless, we found that reducing *pnk‐1* transcript levels by 50%, by either *pnk‐1* RNAi or a deletion mutation upstream of the *pnk‐1* gene (Figure S1A), resulted in a normal lifespan (Figure S1B,C; Table S1). Hence, *pnk‐1* hypomorphic mutants can be exploited for studying the physiological implications of a partial *pnk‐1* deficiency.

**FIGURE 1:**
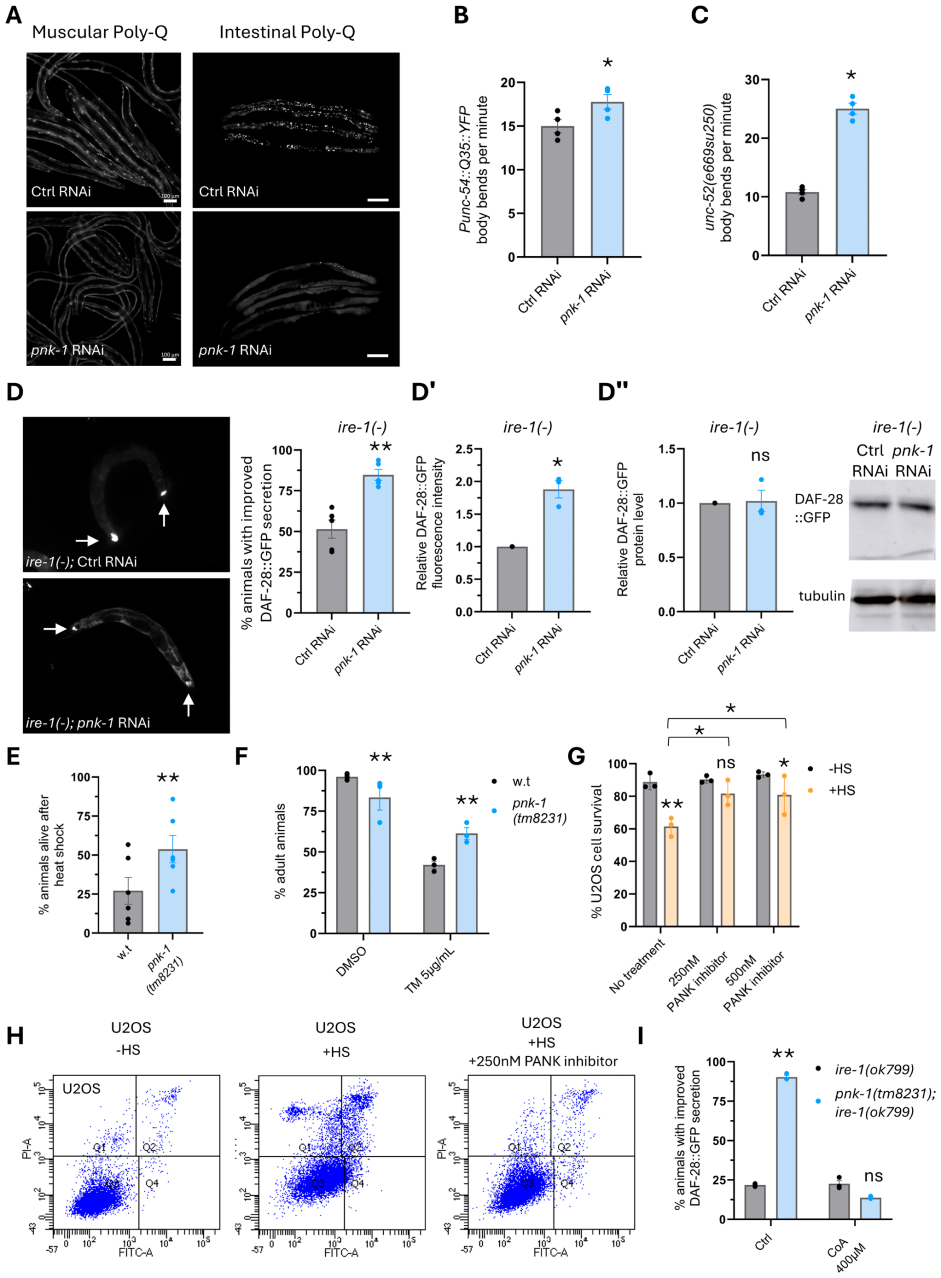
*pnk‐1* deficiency promotes proteostasis. (A) *pnk‐1* RNAi reduced the amount of visible foci in animals expressing muscular PolyQ_35_::YFP or intestinal PolyQ_44_::YFP. Animals were scored on day 5 of adulthood. See foci quantification in Figure S2B. Scale bar: 100 μm. (B) *pnk‐1* RNAi improved the thrashing of animals expressing muscular PolyQ_35_::YFP (*N* = 4, *n* > 90). (C) *pnk‐1* RNAi improved the thrashing of *unc‐52(e669su250)* metastable mutants (*N* = 4, *n* > 80). (D) *pnk‐1* RNAi improved the folding and secretion of a DAF‐28::GFP reporter in *ire‐1(ok799)* mutants. Arrows mark head neurons and intestinal cells that accumulate DAF‐28::GFP protein. *pnk‐1* RNAi increased the fraction of animals with improved DAF‐28::GFP secretion (D, *N* = 5, *n* > 115), increased the overall fluorescence intensity of the DAF‐28::GFP (D′, *N* = 3, *n* > 75), which reflects properly folded GFP, but had no effect on the total DAF‐28::GFP protein level assessed by western blot (D″, *N* = 3), which reflects GFP protein levels irrespective of the folding state of the GFP. (E) *pnk‐1(tm8231)* mutation improved heat‐shock resistance in Day 2 animals (*N* = 6, *n* > 200). (F) *pnk‐1(tm8231)* mutation improved animal development in the presence of the ER stress inducer tunicamycin (*N* = 3, *n* = 300). (G, H) Annexin V/PI flow cytometry analysis of U2OS osteosarcoma cells following 2 h of heat shock. Treatment with the PANK inhibitor increased cell resistance to heat shock (*N* = 3). See Table S2. (I) CoA supplementation suppressed the improved secretion of DAF‐28::GFP in Day 3 *pnk‐1(tm8231); ire‐1(ok799)* mutants (*N* = 3, *n* > 70). Data are shown as mean ± standard error. *N* represents the number of biological repeats, *n* represents the number of animals analyzed per treatment or genetic background. ns, not significant; *p < 0.05; **p < 0.001. Statistical tests: Unpaired student's *t*‐test (B, C); one sample *t*‐test (D′, D″); Cochran–Mantel–Haenszel test (D–F), Two‐way Anova with Tukey's post hoc test (G, I). Comparisons were between w.t. *pnk‐1* and *pnk‐1(tm8231)* mutants per treatment (E, F, I) or relative to the corresponding control RNAi sample (B–D).

We explored how mild *pnk‐1* deficiency affected diseases associated with protein aggregation using a *C. elegans* model of PolyQ expansion diseases, which include Huntington disease, spinobulbar muscular atrophy, dentatorubral‐pallidoluysian atrophy, and several spinocerebellar ataxias (Shao and Diamond 2007). In this model, a reporter gene (YFP) is fused to multiple copies of the amino acid Glutamine (PolyQ), inducing age‐dependent foci formation due to the Poly‐Q tail (Brignull et al. 2006). We examined the effect of *pnk‐1* knockdown by RNAi on the foci of the PolyQ_44_ or PolyQ_35_ reporter in transgenic animals expressing the transgene in their intestine or muscle, respectively. We observed a significant reduction in the number of animals with detectable foci in their muscle and intestine on Day 5 of adulthood following treatment with *pnk‐1* RNAi (Figure 1A; Figures S2A and S2B). However, a filter trap assay comparing YFP aggregate levels in Day 5 animals expressing the Q_35_‐YFP transgene in their muscle indicated that *pnk‐1* RNAi did not decrease Q_35_‐YFP aggregate levels in the muscles compared to control RNAi treated animals (Figure S2C). Thus, in this case, under the given experimental settings, the presence of fewer Q_35_‐YFP foci did not correlate with the level of aggregation. Nevertheless, consistent with the reduced levels of Q_35_‐YFP foci in *pnk‐1* RNAi treated animals, a slight but consistent improvement, ranging between 12% and 26%, in the frequency of body bends per minute was observed in *pnk‐1* RNAi treated animals expressing the PolyQ_35_ transgene in their muscles (Figure 1B), in line with improved muscle homeostasis. Thus, *pnk‐1* RNAi reduced the number of visible YFP‐containing foci in the muscle and preserved muscle function without decreasing the aggregation of the PolyQ35 reporter as detected in the filter retardation assay. One possible explanation for this discrepancy is that there may be different thresholds in the detection of microscopical foci and aggregate entrapment in the filter trap assay.

Notably, unlike the decrease in the intestinal PolyQ foci that were similarly sensitive to *pnk‐1* RNAi (Figure S2A) and the *pnk‐1* hypomorphic mutation (Figure S2D), the decrease in muscular PolyQ foci and improved thrashing were observed only upon *pnk‐1* RNAi treatment (Figure 1B; Figure S2B) and were not observed in the *pnk‐1* mutant (Figure S2E). As *pnk‐1* RNAi and the hypomorphic *pnk‐1* mutation both reduced *pnk‐1* transcript levels to a similar extent in whole body RNA preparations (Figure S1A), we suggest that the discrepancy of their effect on muscular PolyQ foci levels and thrashing may be due to different inter‐tissue local levels of *pnk‐1* expression in response to *pnk‐1* RNAi (Winston et al. 2002) or due to the hypomorphic mutation in the promoter region of *pnk‐1*. Such potential inter‐tissue variation could escape detection when assessing the overall level of the *pnk‐1* transcript.

### 2.2 *pnk‐1* Deficiency Promotes Proteostasis in *C. elegans*

Functional assays which depend on the proper folding of specific proteins offer a simple method for assessing protein folding. Hence, to investigate the impact of the partial loss of expression of *pnk‐1* on protein folding, we assessed the folding efficiency of a metastable protein, using a strain with a temperature‐sensitive *e669su250* mutation in the *unc‐52* gene, as previously described (Ben‐Zvi et al. 2009). This gene encodes a critical component of the basement membrane underlying muscle cells, crucial for proper muscle assembly and muscle function. Thus, misfolded UNC‐52 impairs the thrashing movement of animals in liquid. We found that treatment with *pnk‐1* RNAi more than doubled the frequency of body bends per minute in *unc‐52(e669su250)* mutants on Day 2 of adulthood at the non‐permissive temperature (Figure 1C). An improvement in the frequency of body bends per minute in *unc‐52(e669su250)* mutants was also observed in the presence of the *pnk‐1(tm8231)* mutation (Figure S3A). This suggests that reduced *pnk‐1* levels improve the folding of this metastable protein.

Another way to assess protein folding is to track proteins along the secretory pathway. To this end, we evaluated the effect of *pnk‐1* partial loss on protein secretion using the DAF‐28::GFP secretory model (Safra and Henis‐Korenblit 2014; Safra et al. 2013). In this model, we follow the secretion of a labeled insulin protein (DAF‐28::GFP) expressed in select neurons and intestinal cells in *C. elegans* . Entrapment of the protein within the producing cells indicates a secretory defect, whereas its successful secretion into the body cavity of the animals indicates complete processing of the protein. Furthermore, DAF‐28::GFP secretion allows the differential detection of the properly folded population of DAF‐28::GFP protein (detected by fluorescent DAF‐28::GFP) and the total population of the protein, irrespective of its folding state (detected by Western blotting) (Safra et al. 2013; Safra et al. 2014). To examine whether partial loss of *pnk‐1* expression improves DAF‐28::GFP folding and secretion under chronic stress, we examined DAF‐28::GFP expression pattern and levels in Day 3 animals with a defective ER stress response due to a mutation in the *ire‐1* gene. As previously reported, these proteostasis‐challenged animals fail to secrete the reporter, which remains trapped in the ER of the cells that produce it (Safra et al. 2013). We found that both treatment with *pnk‐1* RNAi and the hypomorphic *pnk‐1(tm8231)* mutation improved the secretion of the reporter in *ire‐1*‐deficient animals, as reflected by the increased proportion of animals with the DAF‐28::GFP detected in their body cavity at the expense of the intestinal cells that produce it (Figure 1D; Figure S3B). Furthermore, *pnk‐1* RNAi significantly increased the fluorescence of the reporter but had no significant effect on total protein levels of the reporter (Figure 1D',D''), indicating an increase in the proportion of properly folded reporter. These results support the notion that partial loss of *pnk‐1* expression improves the folding and processing of the DAF‐28::GFP reporter.

### 2.3 *pnk‐1* Deficiency Promotes Stress Resistance in *C. elegans* and in Mammalian Cells

**FIGURE 2:**
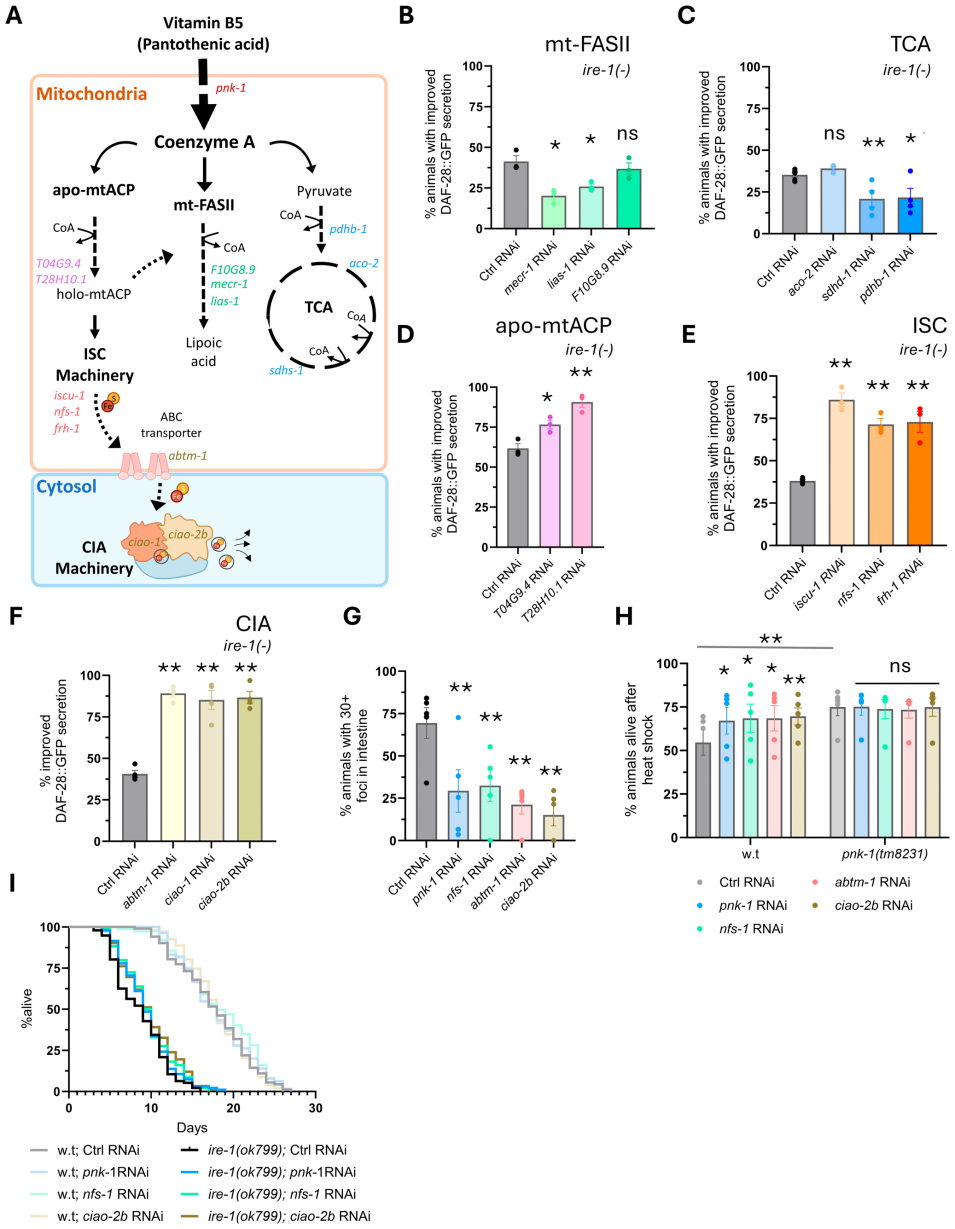
CoA and ISC deficiency act in the same pathway to promote proteostasis. (A) Schematic overview of the central biochemical mitochondrial pathways involving CoA including the mitochondrial fatty‐acid biosynthesis pathway (mt‐FASII, green), the tricarboxylic acid cycle (TCA/Krebs cycle, blue), mitochondrial acyl carrier proteins (mtACP, pink), mitochondrial iron sulfur clusters biogenesis (ISC, orange), and cytosolic iron sulfur cluster assembly (CIA, yellow). (B–F) RNAi knockdown of genes related to mt‐FASII (B) and TCA (C) pathways did not improve DAF‐28::GFP secretion in Day 3 *ire‐1(ok799)* animals. RNAi knockdown of genes related to ACP‐acyl carrier protein (D), iron sulfur clusters (ISC) (E) and cytosolic iron sulfur cluster assembly (CIA) (F) improved DAF‐28::GFP secretion in the *ire‐1(ok799)* animals. (*N* ≥ 3, *n* > 90). See Figure S6A for representative images. (G) RNAi knockdown of *pnk‐1* or ISC‐related genes reduced the number of foci in adult Day 5 animals expressing intestinal PolyQ_44_::YFP (*N* = 5, *n* > 150). See Figure S6B for representative images. (H) ISC‐related genes RNAi increased heat‐shock resistance of Day 2 w.t animals but did not further increase the heat resistance of *pnk‐1(tm8231)* mutants (*N* = 5, *n* > 140). (I) Representative lifespan assay of wild‐type and *ire‐1(−)* mutants (*N* = 3). RNAi knockdown of *pnk‐1* or ISC‐related genes did not extend the lifespan of wild‐type or *ire‐1(ok799)* animals, besides *ciao‐2b* RNAi, which extended the lifespan of *ire‐1(−)* mutants by 15% on average. (*N* = 3). Data are shown as mean ± standard error. *N* represents the number of biological repeats, and *n* represents the number of animals analyzed per RNAi condition. ns, not significant; *p < 0.05; **p < 0.001. Cochran–Mantel–Haenszel test followed by FDR correction compared to the corresponding control RNAi sample (B–H).

Since the partial expression of *pnk‐1* improved the resistance to several chronic proteostasis‐related stresses associated with the expression of metastable proteins, we wondered whether it would also promote animal survival upon exposure to external proteostasis‐related stresses. To evaluate the influence of reduced *pnk‐1* levels on sensitivity to proteostasis‐related stresses, we conducted heat shock stress resistance and tunicamycin resistance experiments with wild‐type and *pnk‐1*‐hypomorphic animals. In the heat shock experiment, wild‐type animals and *pnk‐1(tm8231)* or *pnk‐1(RNAi)* mutants were exposed to heat shock on Day 2 of adulthood, and their survival was assessed 16 h later. In the ER stress experiment, we examined the ability of wild‐type animals and *pnk‐1(tm8231)* or *pnk‐1(RNAi)* mutants to develop from eggs to adults in the presence of the ER stress inducer tunicamycin. Consistent with its ability to improve proteostasis, both *pnk‐1* RNAi and the hypomorphic mutation significantly enhanced both thermotolerance and tunicamycin resistance (Figures 1E,F and 2H; Figure S3C).

Being a key enzyme in CoA biosynthesis, PANK is conserved from bacteria to humans (Leonardi et al. 2005). Hence, we examined whether the inhibition of PANK activity may also promote proteostasis in human cells. To this end, human osteosarcoma U2OS cells were treated with a PANK inhibitor and then exposed to heat shock. Cell necrosis and apoptosis were detected by Annexin V‐FITC/Propidium Iodide (PI) staining. Under these stress conditions, apoptotic and necrotic cells were detected, as reflected by the increase in the proportion of the Annexin V negative/PI positive and Annexin V positive/PI positive cell subpopulations. Strikingly, treating cells with a PANK inhibitor that binds to the ATP–PANK enzyme complex (Sharma et al. 2015) significantly limited the heat‐induced cell death of U2OS cells but had no effect on the viability of the cells that were not exposed to heat shock (Figure 1G,H; Table S2). Furthermore, the heat‐induced cell death of adenocarcinomic human alveolar basal epithelial A549 cells also decreased in the presence of the PANK inhibitor (Sharma et al. 2015) (Figure S4A; Table S2). Similar protection from heat‐induced U2OS cell death was observed upon treating the cells with HoPan, which is a competitive substrate of PANK, and its phosphorylated product Pi‐HoPan is an inhibitor of PPCS, the second enzyme in the CoA biosynthetic pathway (Mostert et al. 2021) (Figure S4B). These results suggest that the inhibition of the CoA biosynthesis pathway can promote heat stress resistance in human cells as well.

### 2.4 CoA Supplementation Negates the Proteostasis Benefits of *pnk‐1* Deficiency

PNK‐1 is the rate‐limiting enzyme in CoA biosynthesis (Leonardi et al. 2005). Hence, we examined whether the proteostasis improvement associated with partial *pnk‐1* deficiency is affected by external supplementation with CoA. To this end, we repeated the acute heat shock survival assay and the chronic DAF‐28::GFP secretion assay with or without CoA supplementation. Remarkably, external supplementation with 400 μM CoA negated the benefits of the *pnk‐1* reduced expression (Figure 1I; Figure S5A). In contrast, the *pnk‐1(tm8231)* hypomorphic mutation improved heat stress resistance and reduced intestinal foci levels when supplemented with 3 mM Vitamin B5 (Figure S5B,C). This indicates that proteostasis improvement resulting from the partial loss of *pnk‐1* expression can be bypassed by CoA supplementation.

The ability of Pantethine supplementation to reverse phenotypes associated with disrupted pantothenate kinase function has been previously observed in Drosophila models of PANK deficiency (Rana et al. 2010). Hence, we examined whether Pantethine supplementation could reverse the *pnk‐1* hypomorphic phenotypes. To this end, we repeated the acute heat shock survival assay and the chronic intestinal PolyQ foci assay with or without Pantethine supplementation. We found that external supplementation with 100 μM Pantethine did not affect the heat stress resistance of *pnk‐1(tm8231)* mutants, nor did it affect the low levels of the PolyQ intestinal foci in *pnk‐1(tm8231)* mutants (Figure S5B,C), suggesting that the limited *pnk‐1* levels in these mutants cannot support effective conversion of Pantethine into CoA. Notably, in wild‐type animals, Pantethine supplementation on its own limited the levels of the PolyQ intestinal foci (Figure S5C). This is consistent with beneficial effects previously associated with Pantethine treatment in the context of counteracting Aβ pathogenesis (Baranger et al. 2019).

### 2.5 *pnk‐1* Deficiency Promotes Proteostasis by Limiting ISC Availability

CoA participates in hundreds of biochemical processes in the cell, many of which take place within the mitochondria (Leonardi et al. 2005). Thus, we examined whether the impairment of mitochondrial pathways, which depend on CoA, promotes proteostasis. To this end, we applied RNAi targeting a selection of candidate genes acting in mitochondrial pathways, which utilize CoA. These included the mitochondrial fatty acid synthesis pathway (*mecr‐1*, *lias‐1*, *F10G8.9*), the Krebs/TCA Cycle (*aco‐2*, *sdhd‐1*, *pdhb‐1*), mitochondrial acyl carrier proteins (ACPs) (*T04G9.4*, *T28H10.1*) and the iron–sulfur cluster (ISC) biogenesis pathway (*iscu‐1*, *nfs‐1*, *frh‐1*) (Figure 2A). We examined whether RNAi treatments of the candidate genes affected the secretion of the secretory reporter DAF‐28::GFP, as observed with *pnk‐1* RNAi (Figure 1D). Interestingly, silencing of *mecr‐1*, *lias‐1*, and *F10G8.9* genes implicated in mitochondrial fatty acid synthesis and silencing of *aco‐2*, *sdhd‐1*, and *pdhb‐1* genes implicated in the Krebs/TCA Cycle did not improve, and in most cases further interfered, with the secretion of the reporter (Figure 2B,C). In contrast, silencing of *T04G9.4* and *T28H10.1* genes encoding mitochondrial acyl carrier proteins (ACP) and silencing of the iron–sulfur cluster (ISC) formation genes *iscu‐1*, *nfs‐1*, and *frh‐1* enhanced the secretion of the reporter (Figure 2D,E). Since ACPs are also an essential subunit which stabilizes the eukaryotic Fe‐S biogenesis complex (Braymer and Lill 2017), it is likely that ACPs and ISC formation genes affect proteostasis by a common mechanism.

ISC are generated within the mitochondria by the mitochondrial ISC complex and are subsequently either utilized within the mitochondria or transported to the cytoplasm (Lill and Freibert 2020). In the cytoplasm, a protein complex known as the cytosolic iron–sulfur cluster machinery assembly (CIA) loads these clusters onto carrier proteins, which transfer them to target proteins outside of the mitochondria (Paul and Lill 2015). Therefore, we explored whether the improvement in proteostasis is also observed upon disruption of the CIA complex (perturbing iron–sulfur dependent activities in the cytoplasm but not affecting those in the mitochondria). To this end, we monitored DAF‐28::GFP secretion upon RNAi silencing of the Fe‐S cluster transporter *abtm‐1*, that facilitates cluster transfer from mitochondria to the cytoplasm, or silencing of the *ciao‐1*, *ciao‐2b* genes, which encode components of the cytoplasmic ISC machinery. Knockdown of all genes implicated in cytoplasmic iron–sulfur cluster metabolism improved the secretion of the DAF‐28::GFP reporter (Figure 2F; Figure S6A). Similarly, silencing of ISC‐related genes, be it in the mitochondria or in the cytoplasm, reduced the foci levels of the PolyQ_44_ intestinal reporter (Figure 2G; Figure S6B) and improved heat shock survival, similarly to *pnk‐1* RNAi treatment (Figure 2H). Thus, deficiency in mitochondria or cytoplasmic ISCs is sufficient for proteostasis improvement.

Finally, since CoA is required for ISC biogenesis (Shi et al. 2021) and since deficiencies in either *pnk‐1* or ISC biogenesis components in the mitochondria or in the cytosol improve proteostasis, we hypothesized that they might operate within the same genetic pathway. To test this hypothesis, we conducted an epistasis experiment, treating *pnk‐1(tm8231)* mutants with RNAi targeting ISC/CIA genes and compared their resistance to heat shock. We found that whereas these RNAi treatments increased heat shock survival of wild‐type animals (Figure 2H, left panel), they did not further increase heat shock survival of *pnk‐1(tm8231)* mutants (Figure 2H, right panel). The lack of an additive effect between *pnk‐1* deficiency and ISC biogenesis deficiencies supports the hypothesis that they promote proteostasis by a common mechanism. Since CoA is required for effective ISC biogenesis, these results imply that *pnk‐1* deficiency promotes proteostasis by limiting ISC biogenesis and specifically ISC availability in the cytoplasm.

### 2.6 *pnk‐1* and ISC Deficiencies Do Not Extend Lifespan

Although proteostasis is not always coupled with longevity, the loss of proteostasis is a fundamental aspect of the aging process, stemming from the malfunction of the cells' protein stress responses and the consequential age‐dependent accumulation of misfolded proteins (Hipp et al. 2019). Since a perturbation to the CoA and ISC biogenesis pathways significantly improved proteostasis under various stress conditions, and since no lifespan benefit was observed in animals with reduced *pnk‐1* expression under normal growth conditions (Figure S1B,C), we postulated that given the proteostasis‐related benefits of *pnk‐1* mutants, a survival benefit might be detected under proteostasis stress conditions. To this end, we examined if *pnk‐1* RNAi affected the lifespan of animals with a perturbed ER stress response due to a mutation in the *ire‐1* gene. Under these stress conditions, no consistent extension of lifespan was observed upon *pnk‐1* or *nfs‐1* RNAi (Figure 2I). A lifespan extension of 14%–18% was observed upon treatment with RNAi targeting the *ciao‐2b* gene, resulting in the dysfunction of the CIA complex (Figure 2I; Table S1). We note that a small lifespan extension has also been reported upon RNAi targeting the mitochondrial iron–sulfur cluster assembly protein component *iscu‐1* (Sheng et al. 2021). Thus, whereas partial impairment of *pnk‐1* and the ISC biosynthetic pathways consistently improved proteostasis, their effect was not coupled to lifespan extension under non‐stress conditions, nor under the examined stress conditions.

### 2.7 Reduced *pnk‐1* Expression Improves Proteostasis in a Chaperone‐Dependent Proteasome/Lysosome Independent Manner

The two major protein quality control mechanisms for improving proteostasis involve protein refolding and clearance of misfolded proteins (Meléndez and Levine 2009; Chen et al. 2011). As such, we examined whether the proteostasis enhancement resulting from reduced expression of *pnk‐1* is dependent on the main protein degradation machinery of the cell (the proteasome or the lysosome) and whether it depends on cellular chaperones (critical for protein refolding, but also for protein solubilization, disaggregation, accumulation in specialized compartments and protein degradation).

**FIGURE 3:**
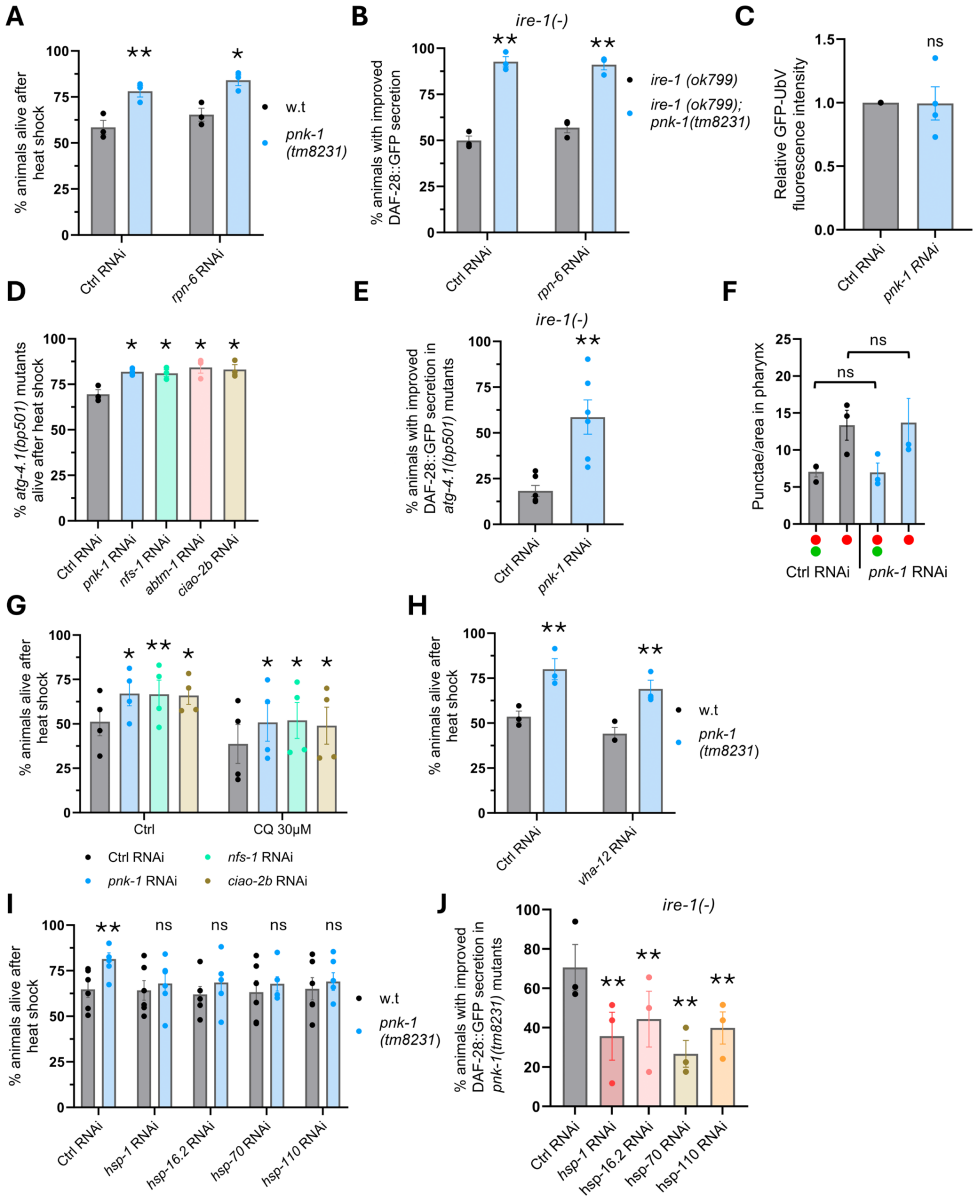
Proteostasis improvement upon interference with CoA and ISC biosynthetic pathways depends on protein chaperones. (A, B) Inhibition of proteasome function by *rpn‐6* RNAi treatment did not affect heat‐shock resistance of *pnk‐1(tm8231)* animals at Day 2 of adulthood (*N* = 3, *n* > 80) (A) nor did it affect DAF‐28::GFP secretion improvement by *pnk‐1(tm8231)* in *ire‐1(ok799)* Day 3 mutants (*N* = 3, *n* > 70) (B). (C) The fluorescence of the GFP‐UbV proteasome substrate reporter did not change upon *pnk‐1* RNAi treatment (*N* = 4, *n* > 90). One sample *t*‐test. (D, G) *pnk‐1* and ISC‐related gene inhibition improved heat‐shock resistance independent of autophagy (*N* = 3, *n* > 90) (D) and independent of chloroquine (CQ) treatment (*N* = 4, *n* > 160) (G). (E) *pnk‐1* RNAi improved DAF‐28::GFP secretion in Day 3 *atg‐4.1(bp501); ire‐1(ok799)* mutants (*N* = 6, *n* > 144). (F) The distribution of autophagosomes and autolysosomes did not change upon *pnk‐1* RNAi treatment (*N* = 3, *n* > 45). Two‐way Anova. (H) Inhibition of lysosome function by *vha‐12* RNAi treatment did not affect heat‐shock resistance of *pnk‐1(tm8231)* animals (*N* = 3, *n* > 105). (I, J) RNAi knockdown of select cytosolic chaperone genes abolished the beneficial effects of *pnk‐1* deficiency on heat resistance (*N* = 6, *n* > 150) (I) and on DAF‐28::GFP secretion in *ire‐1(ok799)* mutants (*N* = 3, *n* > 90) (J). Data are shown as mean ± standard error. *N* represents the number of biological repeats, *n* represents the number of animals analyzed per treatment or genetic background. ns, not significant; *p < 0.05; **p < 0.001. Statistical test was Cochran–Mantel–Haenszel test after FDR correction unless indicated otherwise. Comparisons were between w.t *pnk‐1* and *pnk‐1(tm8231)* mutants per RNAi treatment (A, B, H, I) or relative to the corresponding control RNAi sample (D, E, G, J).

To examine the contribution of the proteasome to the proteostasis of animals with reduced expression of *pnk‐1*, we tested whether the improved heat shock resistance and the improved DAF‐28::GFP secretion of the *pnk‐1* mutants are proteasome dependent. Specifically, DAF‐28::GFP protein secretion and heat shock resistance of *pnk‐1* mutants were compared between animals treated with control RNAi or with RNAi targeting the proteasome regulatory subunit *rpn‐6.1* starting at the L4 stage (earlier treatment of *rpn‐6.1* RNAi is lethal and demonstrated the efficacy of this RNAi treatment). Strikingly, *rpn‐6.1* RNAi treatment did not affect DAF‐28::GFP secretion or heat shock resistance of wild‐type or *pnk‐1* mutant animals (Figure 3A,B). Likewise, no significant change in the fluorescence of an artificial proteasome substrate was observed upon *pnk‐1* RNAi treatment (Figure 3C). Nor did we detect any significant change in the steady state levels of highly ubiquitinated proteins in the *pnk‐1* hypomorphic mutant (Figure S7A). This indicates that proteasome function is not enhanced in *pnk‐1* mutants and that reduced expression of *pnk‐1* improves heat resistance and ER secretory function independent of the proteasome.

Next, we examined whether autophagy is implicated in the improvement of proteostasis upon PNK‐1/ISCs partial loss. First, we examined whether RNAi treatment against *pnk‐1* and ISC‐related genes improves heat shock resistance of autophagy mutants. We found that perturbations in the *pnk‐1*/ISCs pathways increased heat shock survival of *atg‐4.1* and *atg‐9* autophagy mutants (Figure 3D; Figure S7B), as it did for wild‐type animals (Figure 2H). Likewise, RNAi of *pnk‐1* in *atg‐4.1* mutants significantly improved DAF‐28::GFP secretion (Figure 3E) and RNAi silencing of *pnk‐1* and *ciao‐2b* in *atg‐4.1* mutants effectively reduced foci levels in the PolyQ intestine model (Figure S7C). Finally, *pnk‐1* RNAi treatment did not significantly alter the ratio of the autophagosomes and autolysosomes in transgenic animals expressing an *mCherry::gfp::lgg‐1* reporter (Figure 3F). Likewise, only a marginal change in the levels of the autophagy substrate SQST‐1::GFP was observed upon *pnk‐1* RNAi (Figure S7D). These results indicate that macro‐autophagy is not induced by *pnk‐1* RNAi and is not required for the proteostasis improvement by PNK‐1/ISCs partial loss.

Proteins can undergo lysosomal degradation through alternative autophagy pathways such as chaperone‐mediated autophagy and micro‐autophagy (Tekirdag and Cuervo 2018). Thus, we investigated whether *pnk‐1* deficiency‐mediated proteostasis improvement implicates the lysosome. To this end, wild‐type animals were treated with control RNAi or with RNAi against *pnk‐1*/ISC‐related genes in the presence of 30 mM chloroquine (CQ), a lysosomal inhibitor that neutralizes lysosomal acidity and consequently interferes with the degradative activity within the lysosome. The survival of the animals was evaluated 16 h post heat shock treatment. Despite the decreased survival rate of CQ‐treated animals, RNAi against *pnk‐1* or ISC‐related genes increased animal survival even in the presence of CQ (Figure 3G). Similarly, *pnk‐1* mutants survived heat shock better than their wild‐type controls, even upon treatment with RNAi targeting components of the lysosomal v‐ATPase subunits responsible for lysosomal acidification (Figure 3H; Figure S7E). Thus, reduced expression of *pnk‐1* improves heat shock resistance independent of the degradative functions of the lysosome.

Since proteostasis improvement in *pnk‐1* hypomorphs is independent of the proteasome and autophagy, we next explored the possibility that it may rely on improved protein folding rather than protein degradation. Protein refolding is carried out by dedicated chaperone proteins. To test whether chaperone‐based protein processing might be required for proteostasis improvement upon reduction of *pnk‐1* expression, we tested for an effect of RNAi against candidate genes encoding chaperones (14 cytosolic chaperones and 5 ER resident chaperones) on the survival of wild‐type and *pnk‐1(tm8231)* mutants. Strikingly, RNAi treatment against 9/14 of the cytosolic chaperones and against 4/5 of the ER resident chaperones compromised the relative survival of *pnk‐1* mutants compared to the matched RNAi‐treated wild‐type animals (Table S3). We confirmed that RNAi treatment against four of the chaperones identified in the mini‐chaperone candidate screen indeed compromised heat‐shock survival of *pnk‐1* mutants (Figure 3I). The finding that many chaperone proteins contribute to the heat shock resistance of *pnk‐1* mutants, along with the findings that reduced *pnk‐1* expression improves the proportion of properly folded DAF‐28::GFP insulin reporter (Figure 1D) and improves the folding of a metastable protein (Figure 1C; Figure S3A), all implicate chaperone‐mediated protein folding as a prime mechanism that improves proteostasis in these mutants.

### 2.8 HLH‐30/TFEB Binds to the Promoter of Many Chaperone Genes

**FIGURE 4:**
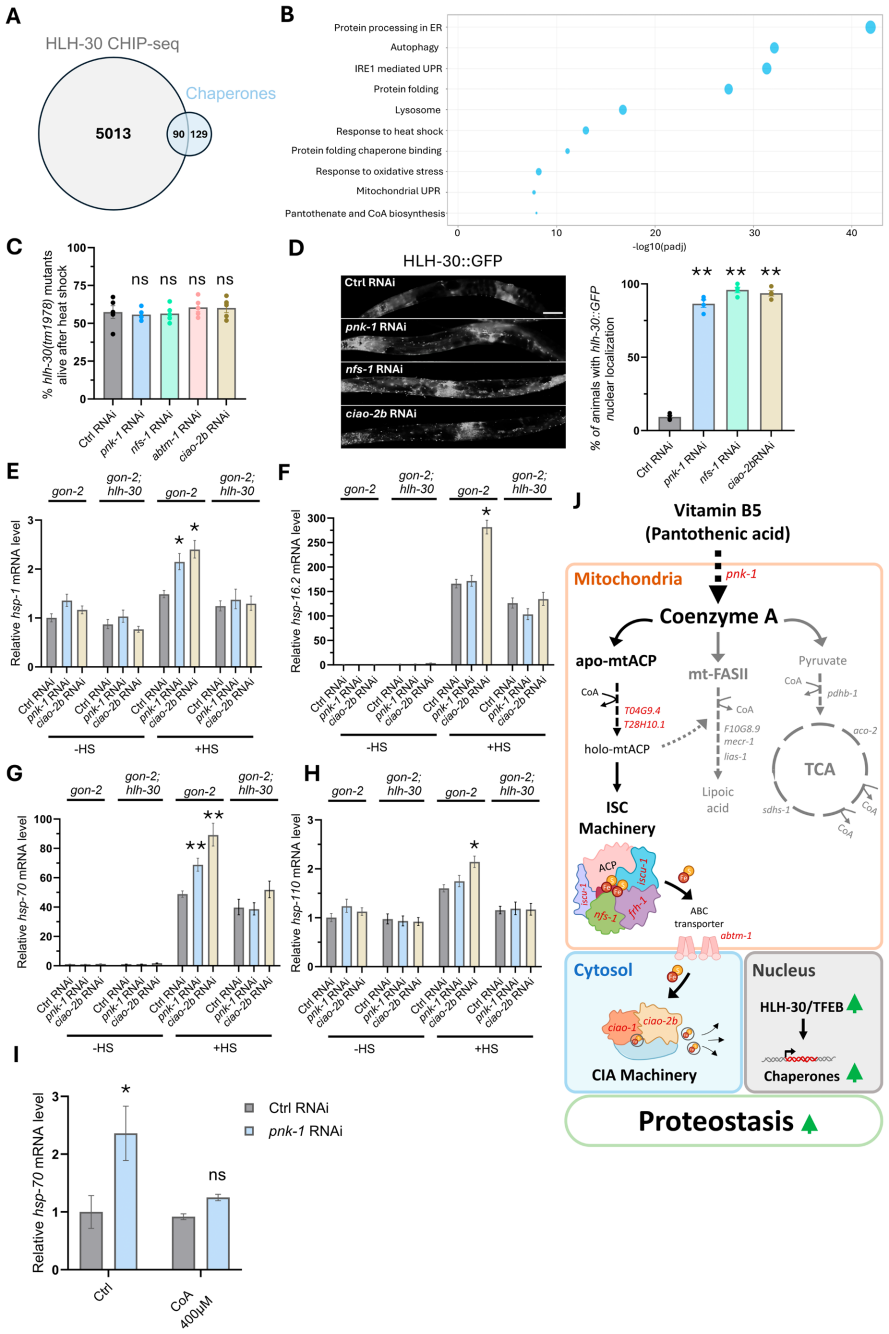
HLH‐30 promotes chaperone expression under CoA and ISC mild deficiency. (A) Venn diagram depicting an overlap between 5103 potential HLH‐30 CHIP‐seq defined targets (not including linc genes) and 219 *C. elegans* chaperone and co‐chaperone genes (gray and blue, respectively, Table S4). Nearly 40% of the chaperone genes overlapped with the group of potential HLH‐30 CHIP‐seq defined targets. (B) Gene set enrichment analysis of the potential HLH‐30 CHIP‐seq defined targets revealed enrichment in genes related to protein quality control, protein folding, chaperones, response to heat, and the ER and mitochondria unfolded protein responses, lysosome, and autophagy‐related genes. Enrichment of pantothenate and CoA biosynthesis genes was also observed. (C) *hlh‐30(tm1978)* mutation suppressed the benefits of *pnk‐1* and ISC‐related genes inhibition in the heat‐shock resistance test. (*N* = 5, *n* > 175). (D) *pnk‐1* and ISC‐related genes knockdown increased the nuclear translocation of an HLH‐30::GFP transgene in Day 3 animals (*N* = 4, *n* > 100). Scale bar: 100 μm. (E–H) Transcript levels of cytosolic chaperones were examined by qRT‐PCR in day 2 adult *gon‐2(q388)* and *gon‐2(q388); hlh‐30(tm1978)* double mutants treated with RNAi against control, *pnk‐1*, or *ciao‐2b* (*N* = 4). In all cases, RNAi treatment against *pnk‐1* and/or *ciao‐2b* further enhanced the expression of the indicated chaperone genes upon heat shock. (I) The improved induction of *hsp‐70* transcripts in response to heat stress upon *pnk‐1* RNAi treatment was suppressed by CoA supplementation (*N* = 3). (J) Summary model: CoA and/or ISC deficiencies improve proteostasis via activation of the conserved HLH‐30/TFEB transcription factor. HLH‐30/TFEB promotes the expression of a wide range of protein quality control genes, including chaperones, which are critical for the proteostasis improvement. Data are shown as mean ± standard error. *N* represents the number of biological repeats, *n* represents the number of animals analyzed per treatment or genetic background. ns, not significant; *p < 0.05; **p < 0.001. Statistical tests: Cochran–Mantel–Haenszel test followed by FDR correction (C, D), Two‐Way Anova followed by Tukey's post hoc analysis (E–I). Comparisons were relative to the corresponding control RNAi sample.

HLH‐30/TFEB serves as a pivotal transcriptional regulator of autophagy and lysosome biogenesis (Lapierre et al. 2013). Nevertheless, we noticed that 10/13 of the chaperones that contributed to the heat shock survival of *pnk‐1* mutants contained an upstream HLH‐30 binding site, as defined by published HLH‐30 CHIP‐seq data (Gerstein et al. 2010). To further explore the possibility that HLH‐30 is a major regulator of chaperone genes, we analyzed the overlap between the complete set of 5103 potential HLH‐30 CHIP‐seq‐defined targets (Gerstein et al. 2010; Price et al. 2023) and the complete set of 219 *C. elegans* chaperone and co‐chaperone genes (Brehme et al. 2014). We identified 90 overlapping genes among these two gene groups, which is significantly higher than expected by chance (Representation factor: 1.6, associated probability *p* < 3.069e‐07) (Figure 4A; Table S4). Likewise, unbiased gene set enrichment analysis (EasyGSEA, Evitta (Cheng et al. 2021)) of the potential HLH‐30 CHIP‐seq‐defined targets revealed enrichment in many protein quality control‐related gene sets, including chaperones, in addition to lysosome and autophagy‐related genes, which are most commonly associated with this transcription factor. Specifically, these included genes related to protein folding, the heat stress response, and the ER stress and mitochondria unfolded protein responses (Figure 4B; Table S5). Interestingly, the pantothenate and CoA biosynthesis pathway genes, including *pnk‐1* itself, were also over‐represented in the HLH‐30 CHIP gene set, suggesting that the HLH‐30 transcription factor modulates CoA biosynthesis.

### 2.9 HLH‐30/TFEB Is Required for Proteostasis Improvement Upon Interference With the CoA/ISC Biosynthetic Pathways

To examine whether a deficiency in the *pnk‐1* pathway leads to improved proteostasis via HLH‐30/TFEB, we subjected *hlh‐30* mutants to *pnk‐1* RNAi and assessed their survival to heat shock. Unlike wild‐type animals, whose heat shock resistance was improved by *pnk‐1* RNAi or RNAi silencing of ISC‐related genes (Figure 2H), no improvement in survival by these RNAi treatments was observed in *hlh‐30* mutants (Figure 4C). Additionally, in the DAF‐28::GFP secretory assay, the absence of *hlh‐30* completely repressed the positive impact of *pnk‐1* RNAi (Figure S8A). Furthermore, increased nuclear translocation of an HLH‐30::GFP transgene was observed in Day 3 animals exposed to RNAi against *pnk‐1* or ISC‐related genes (Figure 4D). These findings demonstrate that the TFEB transcription factor HLH‐30 is activated by deficiencies in CoA/ISCs biogenesis, and its activity is required for improving proteostasis under these conditions.

Stress responses in general, and proteostasis stress responses in particular, usually implicate stress‐related transcription factors that promote adaptive transcriptional reprogramming. Hence, we examined the contribution of four well‐characterized stress‐related transcription factors to the proteostasis changes associated with the reduced expression of *pnk‐1*. Specifically, we focused on the transcription factors DAF‐16, SKN‐1, HSF‐1, and PQM‐1, which have been implicated in the regulation of aging, proteostasis, and stress resistance (Grushko et al. 2021; Murphy et al. 2003; Morimoto 2011; Tepper et al. 2013). To examine whether any of these transcription factors are needed for the proteostasis modulation by *pnk‐1* reduced expression, we treated *pnk‐1(tm8231*) mutants with RNAi against each of the transcription factors and examined their effect on heat shock resistance and PolyQ foci amount in the intestine. RNAi efficiency was confirmed by qRT‐PCR (Figure S8B). We found that the increased heat stress resistance of *pnk‐1* mutants was dependent on *skn‐1*, *hsf‐1*, and *pqm‐1* (Figure S8C), whereas the reduced levels of PolyQ foci in the intestine of *pnk‐1* mutants were dependent on *skn‐1* and *pqm‐1* (Figure S8D). Thus, HLH‐30 is not the only transcription factor contributing to proteostasis maintenance in *pnk‐1* hypomorphic mutants.

### 2.10 HLH‐30/TFEB Potentiates Chaperone Expression in Heat‐Stressed CoA/ISC Deficient Animals

Since *hlh‐30* is important for proteostasis improvement in *pnk‐1* mutants and given that this resilience to proteostasis stress was chaperone dependent but lysosome independent, we next examined whether transcripts of cytosolic chaperones with putative HLH‐30 binding sites were upregulated in response to reduced expression of *pnk‐1* or of genes of the ISC pathway, and whether this may be mediated by the HLH‐30/TFEB transcription factor. To this end, chaperone transcript levels were examined by qRT‐PCR in *gon‐2(q388)* and *gon‐2(q388); hlh‐30(tm1978)* double mutants treated with RNAi against control, *pnk‐1*, or *ciao‐2b* (Figure 4E–H; Table S6). The *gon‐2(q388)* temperature‐sensitive mutation impairs gonad development, ensuring that the detected transcripts represent the stress response of the mothers rather than that of the embryos/germline. Importantly, *gon‐2(q388)* mutation had no impact on the heat‐shock benefits conferred by the *pnk‐1*/*ciao‐2b* RNAi (Figure S8E). Strikingly, *pnk‐1* RNAi enhanced the induction in the transcript levels of two out of four cytosolic chaperones examined upon heat shock (*hsp‐1* and *hsp‐70*, Figure 4E,G). The heat‐shock induction of the transcripts of all four cytosolic chaperones was more pronounced upon treatment with *ciao‐2b* RNAi (Figure 4E–H). In the case of *hsp‐1* and *hsp‐110*, the heat shock‐induced increase in their transcript levels was only observed upon knockdown of *pnk‐1 and/or ciao‐2b* (Figure 4E,H). For *hsp‐16.2* and *hsp‐70* genes, knockdown of *pnk‐1* and/or *ciao‐2b* further increased the levels of their transcripts, beyond the basal induction observed upon heat shock (Figure 4F,G). In all cases, the *pnk‐1/ciao‐2b* RNAi‐mediated increase in chaperone transcript levels was only observed upon heat stress and was *hlh‐30*‐dependent (Figure 4E–H; Table S6). Furthermore, the potentiated expression of the *hsp‐70* transcript was reversed upon CoA supplementation (Figure 4I). These results suggest that HLH‐30 promotes the transcription of cytosolic chaperones to boost proteostasis resistance upon a partial deficiency in the CoA/ISC biogenesis pathways.

## 3 Discussion

Loss of function mutations in PANK limit CoA biosynthesis and are usually associated with neurodegenerative disorders such as Pantothenate kinase‐associated neurodegeneration (PKAN), dystonia, Parkinson's disease, and Alzheimer's disease. However, less is known about the consequences of mild deficiencies in *PANK* homologs.

In this study, we employed the model organism *C. elegans* to investigate the consequences of a partial reduction in the expression of *pnk‐1*. We applied two models for the mild deficiency in *pnk‐1*: silencing of *pnk‐1* by RNAi and a mutation in the putative promoter region of *pnk‐1*. Both approaches led to a two‐fold reduction in the levels of *pnk‐1* transcripts. Unlike the characteristic pathology of a complete loss of function in *pnk‐1*, hypomorphic *pnk‐1* mutants had a normal lifespan and were resistant to a wide range of proteostasis challenges. These included stress in the ER and in the cytosol as well as acute stresses, such as heat shock, and chronic stresses, as in the case of animals with a defective ER stress response due to a mutation in the *ire‐1* gene and the *C. elegans* PolyQ44 intestinal foci disease model. Furthermore, CoA supplementation reversed the proteostasis effects of animals with reduced *pnk‐1* expression. Finally, the inhibition of key enzymes in the CoA biogenesis pathway protected from heat stress also in two human cell lines, demonstrating the potential conservation and cell‐autonomous nature of this proteostasis promoting pathway. Thus, diverse manipulations to the CoA biosynthesis pathway in a variety of experimental systems result in improved proteostasis.

The only exception in which *pnk‐1* knockdown did not improve proteostasis was the lack of effect of the hypomorphic mutation on the muscular PolyQ35 foci *C. elegans* model, where *pnk‐1* RNAi reduced foci amount and improved muscle function, but the *tm8231* mutant did not. Given the consistent proteostasis‐promoting effects of the *pnk‐1* hypomorphic mutation in many other proteostasis challenges, and given the proteostasis‐promoting effect of *pnk‐1* RNAi on muscular PolyQ35 foci, we suggest that the lack of effect of the *pnk‐1* mutation in this system may be due to tissue‐specific variation in the suppression efficiency of *pnk‐1* transcript expression, due to the deletion mutation within the promoter region of the *pnk‐1* gene.

Proteostasis improvement by *pnk‐1* knockdown was suppressed by external supplementation with CoA, the end product of the CoA biogenesis pathway, but was not suppressed by supplementation with vitamin B5 or Pantethine, the direct and indirect precursors of this pathway. This is consistent with the possibility that limitation of CoA biosynthesis triggers the observed proteostasis improvement. Nevertheless, since the *pnk‐1* mutants analyzed were hypomorphic mutants and not complete loss‐of‐function mutants, and since CoA levels were not directly assessed, we cannot rule out the possibility that CoA production was compensated for by other salvage pathways (Srinivasan et al. 2015; Sibon and Strauss 2016) and that other consequences of a mild deficiency in PANK levels promote proteostasis.

Among the many CoA‐dependent events in the mitochondria (Leonardi et al. 2005), knockdown of the ISC machinery also had a robust proteostasis improvement effect. Epistasis experiments indicated that *pnk‐1* reduced expression and knockdown of ISC‐related genes both improve proteostasis by a common mechanism. This suggests that reduced *pnk‐1* levels may promote proteostasis by limiting ISC formation, which in turn enhances proteostasis (Figure 4J). Interestingly, feeding *C. elegans* bacteria with aberrant Fe‐S cluster biogenesis also enhances the protein stress tolerance of the *C. elegans* host (Bhat et al. 2024), suggesting that ISC deficiency can be controlled through the diet. Since the knockdown of the ISC transporter responsible for exporting ISCs from the mitochondria to the cytosol and knockdown of proteins that load the ISCs onto target cytosolic Fe‐S proteins also improved proteostasis, this uncouples the relationship between mitochondrial and non‐mitochondrial Fe‐S‐related functions. Hence, they implicate cytosolic/nuclear Fe‐S proteins rather than mitochondrial Fe‐S proteins in the regulation of proteostasis.

At the mechanistic level, proteostasis improvement by the deficiencies in CoA/ISC biosynthesis pathways are dependent on chaperones, yet independent of protein quality control mechanisms related to protein degradation such as the proteasome and the lysosome. This suggests that the chaperones that promote proteostasis under these conditions do so by supporting the refolding of misfolded proteins or by altering the solubilization state of the misfolded proteins, rather than by clearing these aberrant proteins via the cellular degradation machineries (Kim et al. 2013). Interestingly, although hundreds of chaperones are encoded in the genome, the improved resistance to proteostasis challenges was lost upon silencing of individual chaperones. This may reflect the lack of redundancy among the various chaperones, which may serve different cellular functions and may have different substrate preferences and thus support different client proteins in the context of proteostasis challenges.

Proteostasis improvement by perturbations in the CoA biosynthetic pathway were dependent on different combinations of stress‐related transcription factors. We focused on the contribution of the transcription factor HLH‐30/TFEB homolog, which translocated to the nucleus upon interference with the CoA/ISC biosynthetic pathways and was essential for improved heat stress resistance. Interestingly, the CoA biosynthesis pathway itself is overrepresented among the HLH‐30 target genes, and TFEB‐dependent increase in CoA biosynthesis has been reported in response to sulfur amino acid metabolism (Matye et al. 2022), constituting a putative negative feedback loop between sulfur and CoA availability and TFEB.

HLH‐30/TFEB is best known for its crucial role in lysosome biogenesis and autophagy (Lin et al. 2018; Settembre et al. 2011), and as an essential transcription factor required and sufficient for most longevity pathways in *C. elegans* (Lapierre et al. 2013). Nevertheless, neither lysosome acidity nor autophagy are required for the improved proteostasis upon CoA/ISC depletion. Instead, the improved proteostasis relies on a chaperone‐mediated proteostasis shield, mediated by individual chaperones whose transcription is potentiated under proteostasis‐challenging conditions in an HLH‐30‐dependent manner. Indeed, analysis of HLH‐30 target genes identified in ChIP experiments reveals that many protein quality control related gene sets, including chaperones and unfolded protein response genes alongside the classical lysosome and autophagy‐related genes, are enriched within the potential target genes of the HLH‐30 transcription factor. This raises the notion that the pro‐longevity (Lapierre et al. 2013) and pro‐proteostasis (Lin et al. 2018) properties of HLH‐30 may be attributed to the rewiring of the entire protein quality control network. Importantly, human TFEB has also been identified as a component of the integrated stress response (Martina et al. 2016), demonstrating its importance beyond autophagy and the lysosomal pathways.

Altogether, HLH‐30/TFEB controls a wide range of protein quality control mechanisms, implying it is an evolutionarily conserved key longevity and proteostasis‐promoting transcription factor, which induces the expression of chaperone and stress response genes alongside autophagy/lysosome genes, all of which may contribute to improved proteostasis and health span. These results highlight TFEB and its upstream regulators as potential therapeutic targets in proteostasis‐related diseases.

## 4 Methods

### 4.1 *C. elegans* Strains Used in This Study

**Table:** 

Strain	Genotype	Source
N2	Wild type	*CGC*
HE250	*unc‐52*(*e669su250*)	*CGC*
SHK649	*pnk‐1*(*tm8231*)	*NBRP strain, out crossed X4*
JIN1375	*hlh‐30*(*tm1978*)	*CGC*
HZ1685	*atg‐4.1*(*bp501*)	*CGC*
SHK578	*atg‐9*(*bp564*)	*CGC strain, out crossed X3*
MAH240	*Phlh‐30*::HLH‐30::GFP + *rol*‐*6(su1006)*	*CGC*
SHK11	*ire‐1(ok799)*; svIs69 [*Pdaf*‐*28*::DAF28::GFP]	*Described in* (Safra et al. 2013)
SHK626	*ire‐1(ok799)*; *svIs69* [*Pdaf*‐*28*::DAF28::GFP]; *pnk‐1*(*tm8231*)	*New strain*
SHK580	*ire‐1(ok799); svIs69* [*Pdaf*‐*28*::DAF28::GFP]; *hlh‐30*(*tm1978*)	*New strain*
SHK494	*ire‐1 (ok799); svIs69* [*Pdaf*‐*28*::DAF28::GFP]; *atg‐4.1*(*bp501*)	*New strain*
QU255	*Is*[*PSQST‐1*::SQST‐1::GFP *+ unc*‐*76*]	*Gift from Prof. Alicia Meléndez*
CF2253	*gon‐2(q388)*	*Described in* (Sun and Lambie 1997)
SHK748	*hlh‐30(tm1978); gon‐2(q388)*	*New strain*
AM738	rmIs297 [*Pvha‐6*::Q_44_::YFP; *rol*‐*6(su1006)*]	*Gift from Prof. Anat Ben‐Zvi*
AM140	*rmIs132* [*Punc‐54*::Q_35_::YFP]	*CGC*
MAH215	*sqIs11* [*lgg‐1p::mCherry::GFP::lgg*‐*1 + rol‐6*].	*CGC*
PP563	unc‐119(ed4)III; hhls64[unc‐119(+);sur‐5::UbV‐GFP]III	*Gift from Prof. Hoppe Thorsten*
SHK789	*pnk‐1*(*tm8231*); *unc‐52(e669su250)*	*New strain*
SHK707	*pnk‐1(tm8231*); rmIs297[*Pvha*‐*6*::Q_44_::YFP; *rol‐6(su1006*)]	*New strain*
SHK790	*pnk‐1(tm8231*); *rmIs132* [*unc*‐*54p*::*Q35*::*YFP*]	*New strain*

### 4.2 Worm Maintenance

Strains were maintained at 20°C using standard *C. elegans* methods. Nematode Growth Media (NGM) agar plates were seeded with *E. coli* strain OP50 or with HT115 bacteria for RNAi experiments. For the supplementation experiment, regular NGM plates served as a control, and treatment plates were supplemented with 400 μM Coenzyme A (Sigma‐Aldrich, C4780), 3 Mm Vitamin B5 (Sigma‐Aldrich, P5155) or 100 μM D‐pantethine (MCE, HY‐B1028).

### 4.3 RNA Interference

HT115 bacteria expressing dsRNA were cultured overnight in LB containing 10 μg/mL tetracycline and 100 μg/mL ampicillin. Before seeding, IPTG was added to the bacteria medium to a final concentration of 20 μM. 500 μL bacteria were seeded on NGM plates containing 2 mM IPTG and 0.05 mg/mL carbenicillin. RNAi clone identity was verified by sequencing. Eggs were placed on fresh plates (2 days after bacteria seeding) and synchronized at L4. *pnk‐1* RNAi expressing HT115 bacteria was obtained from the Julie Ahringer RNAi library. HT115 RNAi bacteria containing pAD12 (from Cynthia Kenyon lab, addgene plasmid # 34832) was used as control RNAi. A list of RNAi clones used in this study is provided in Table S7.

### 4.4 DAF‐28::GFP Secretion Assay

Eggs of *ire‐1(ok799)* animals expressing a *Pdaf‐28::daf‐28::gfp* transgene were grown at 20°C on NGM or RNAi plates until day 3 of adulthood. 3‐day‐old transgenic animals were anesthetized on 2% agarose pads containing 2 mM levamisole (Sigma, L9756). The DAF‐28::GFP expression pattern was examined in at least 30 animals on Day 3 of adulthood. Animals in which the transgene was detected within the body cavity of the animals were scored as animals with improved DAF‐28::GFP secretion. Animals in which the transgene was trapped within the producing cells in the posterior intestine were scored as animals with a secretory defect.

### 4.5 Thrashing Test

*unc‐52(e669su250)* eggs were incubated at 20°C until L4. Age‐synchronized L4 *unc‐52(e669su250)* animals were shifted to 25°C until day 2 of adulthood. On Day 2 of adulthood, animals were placed in 96 wells containing M9 buffer. After a 10 s adjustment period, each animal was monitored and scored for trashing over a 20 s period. Values are presented as body bends per minute.

### 4.6 Tunicamycin Resistance Assay

Eggs were placed on fresh plates containing DMSO or 5 μg/mL Tunicamycin (Sigma‐Aldrich, 654,380). After 3 days, the number of animals that developed to the L4 stage/adult stage was scored.

### 4.7 Heat Shock Survival Assay

Age‐synchronized animals were grown at 25°C until Day 2 of adulthood. On Day 2 of adulthood, animals were subjected to a 37°C heat shock for 5.5 h and recovered at 25°C overnight (~16 h). Animals that failed to move in response to a gentle touch with a metal pick were scored as dead.

For external supplement experiments, eggs were grown at 25°C until Day 2 of adulthood on regular NGM plates, which served as a control, or on plates supplemented with 400 μM Coenzyme A (Sigma‐Aldrich, C4780), 3mM Vitamin B5 (Sigma‐Aldrich, P5155) or 100 μM D‐Pantethine (MCE, HY‐B1028).

### 4.8 Chloroquine Treatment

Eggs were grown at 25°C until Day 2 of adulthood on plates containing 30 μM Chloroquine Diphosphate Salt (Sigma, C6628). On Day 2 of adulthood, animals were subjected to a 37°C heat shock for 5.5 h and recovered at 25°C overnight. Animals that failed to move in response to a gentle touch with a metal pick were scored as dead.

### 4.9 Quantification of Autophagic Vesicles

Quantification of autophagic vesicles was performed as previously described (Chang et al. 2017). In short, *lgg‐1p::mCherry::gfp::lgg‐1* animals were grown on control or *pnk‐1* RNAi until Day 3 of adulthood. Animals were prepared on agarose pads in M9 medium containing 0.1% NaN3. Imaging was performed using a Yokogawa CSU‐W1 Spinning Field Scanning Confocal System. Z‐stack images were captured at 0.6 μm slice intervals using a 40x objective. Positive puncta for GFP::LGG‐1 (GFP) and mCherry::GFP::LGG‐1 (mCherry/GFP or mCherry only) were scored, focusing on the center of the posterior pharyngeal bulb within a 1 μm region, resulting in three planes of 0.6 μm each.

### 4.10 qRT‐PCR

To measure the relative levels of *pnk‐1* transcript, w.t and *pnk‐1(tm8231)* eggs were raised on NGM plates at 20°C until Day 3 of adulthood. On Day 3 of adulthood, synchronized animals were collected for RNA extraction. To check the relative levels of select chaperone transcripts, w.t or *hlh‐30(tm1978)* animals were raised on control, *pnk‐1*, or *ciao‐2b* RNAi at 25°C from eggs until Day 2 of adulthood. On Day 2, animals were exposed to 37°C heat shock for 1 h and recovered at 25°C for 1 h. Then, animals were collected for RNA extraction. Total RNA was extracted with TRIzol reagent (Ambion, 15596026) and treated with RNase‐free DNase I (Thermo scientific, EN0521). Reverse transcription was carried out using the qScript cDNA Synthesis Kit (Quantabio 66,225,515). Real‐time PCR was done using Blue Mix Hi‐Rox SYBR GREEN (PCR Biosystems, Pb20‐16‐05) in a StepOnePlus Real‐Time PCR System. Transcript levels were analyzed by the ΔΔCT method. Transcript levels of *ama‐1* were used for normalization. Each sample was run in triplicates, and four independent biological samples were analyzed. P‐values were calculated using a One Sample *t*‐test for *pnk‐1* mRNA. For chaperones transcripts, *p*‐values were calculated using a Two Way Anova followed by Tukey Post Hoc.

Primers used for qPCR are detailed in Table S8.

### 4.11 Lifespan

Eggs were placed on plates seeded with the RNAi bacteria of interest and were continuously transferred to freshly seeded RNAi plates. Lifespan was scored every 1–2 days. Related lifespans were performed concurrently to minimize variability. In all experiments, lifespan was scored as of the L4 stage, which was set as *t* = 0. Animals that ruptured or crawled off the plates were included in the lifespan analysis as censored worms. The SPSS program was used to determine the means and the P values. P values were calculated using the Log Rank (Mantel‐Cox) and Breslow (Generalized Wilcoxon) methods.

### 4.12 Western Blot

100 day‐3 animals were boiled in protein sample buffer containing 2% SDS. Proteins were separated using standard PAGE separation, transferred to a nitrocellulose membrane, and detected by western blotting using anti‐tubulin (Sigma, T5168, 1∶1000), anti‐GFP (Roche, 11814460001, 1:1000) and anti‐ubiquitin (P4D1) (Santa Cruz, sc‐8017, 1:500) antibodies.

### 4.13 Filter Retardation Assay

Filter retardation assay was performed as previously described (Sin et al. 2018). In short, ~2000 day 2 worms raised from egg at 25° were collected with PBS buffer and worm pellets were frozen with liquid nitrogen. Frozen worm pellets were thawed on ice and worm extracts were generated by glass bead disruption in an equal volume of FTA sample buffer (10 mM Tris‐Cl pH 8.0, 150 mM NaCl, 2% SDS) with protease inhibitors. After beating, samples were spun at 8000 g for 5 min to remove beads and debris, and protein concentration was determined by Bradford assay. Approximately 100 μg of protein extract was supplemented with FTA sample buffer and DTT and heated at 98°C for 5 min prior to loading onto a 0.22 μm cellulose acetate membrane assembled in a slot‐blot apparatus (Bio‐Rad). Samples were filtered through the cellulose acetate membrane, which was washed with 0.2% SDS FTA buffer (10 mM Tris‐Cl pH 8.0, 150 mM NaCl, 0.1% SDS). The retained PolyQ‐expanded proteins were assessed by immunoblotting with anti‐GFP antibodies (Roche, 11814460001, 1:1000). Extracts were also analyzed by SDS–PAGE to determine comparative protein levels based on immunoblotting with anti‐tubulin antibodies (Sigma, T5168, 1∶1000). Antibody binding was visualized with an ECL kit.

### 4.14 Fluorescence Microscopy and Quantification

To follow expression of fluorescent transgenic markers, animals were anesthetized on 2% agarose pads containing 2 mM levamisole. Images were taken with a CCD digital camera using a Nikon 90i fluorescence microscope. For each trial, exposure time was calibrated to minimize the number of saturated pixels and was kept constant throughout the experiment unless indicated otherwise. The NIS element software was used to quantify mean or sum fluorescence intensity of manually selected regions of interest encompassing individual animals.

### 4.15 Cell Culture

Human U2OS cells (ATCC) were maintained in low glucose DMEM (Biological Industries, Beit‐Haemek, Israel) supplemented with 10% fetal bovine serum (FBS; HyClone Laboratories, Logan, UT). Human A549 cells (ATCC) were maintained in high glucose DMEM (Gibco), supplemented with 10% fetal bovine serum. Cells were seeded in 6 well plates to a density of 60%–70%. After 24 h, cells were treated with PANK inhibitor (Sigma, 537983, 250 nM or 500 nM) and HoPan inhibitor (MCE, HY‐139729). DMSO was used as a solvent control for the PANK inhibitor. 16 h following the treatment, cells were exposed to 2 h heat shock at 42°–43°. Immediately thereafter, double staining with Annexin V‐FITC and PI was performed using an Annexin V‐FITC Apoptosis Kit (Bio Vision K101‐100‐3). Flow cytometry analysis was performed on a BD LSR Fortessa Cell Analyzer. Cell viability was determined by the population negative for annexin V and PI.

### 4.16 Statistical Analysis

All experimental results are presented as the mean of at least three independent experiments. All error bars show the SEM, unless otherwise stated. Statistical significance was determined by using statistical tests as indicated in the figure legends and in Table S9. Statistical *p*‐values of < 0.05 were considered significant. Data were analyzed using SPSS. Graphs were created using GraphPad Prism software.

## Author Contributions

Rewayd Shalash, Sivan Henis‐Korenblit: conceptualization. **Rewayd Shalash**, **Dror Michael Solomon**, **Mor Levi‐Ferber**, **Sivan Henis‐Korenblit**, **Matan Yosef Avivi**, **Hagit Hauschner:** methodology. **Rewayd Shalash**, **Dror Michael Solomon**, **Mor Levi‐Ferber**, **Henrik von Chrzanowski**, **Mohammad Khaled Atrash**, **Barak Nakar**, **Aviya Swisa:** investigation. **Rewayd Shalash**, **Mor Levi‐Ferber**, **Yaron Shav‐Tal**, **Sivan Henis‐Korenblit:** writing – original draft. **Alicia Meléndez**, **Yaron Shav‐Tal**, **Sivan Henis‐Korenblit:** supervision. **Sivan Henis‐Korenblit**, **Alicia Meléndez**, **Yaron Shav‐Tal:** funding acquisition.

## Conflicts of Interest

The authors declare no conflicts of interest.

## Supporting information


Data S1.

**Table S1.** The effect of *pnk‐1*/ISCs partial deficiency on lifespan in *C. elegans* .

**Table S2.** The effect of inhibition of enzymes of the CoA biosynthesis pathway on heat shock resistance of human cell lines.

**Table S3.** Chaperone RNAi candidate screen.

**Table S4.** Data sets of potential HLH‐30 CHIP‐seq defined targets and chaperone and co‐chaperone genes.

**Table S5.** Gene set enrichment analysis of the potential HLH‐30 CHIP‐seq targets.

**Table S6.** Cytosolic chaperones qRT‐PCR data.

**Table S7.** RNAi clone data.

**Table S8.** qRT‐PCR primers.

**Table S9.** Statistics.

## Data Availability

All relevant data can be found within the article and its Supporting Information.
