# Supplementary material for: HLH‐30/TFEB Rewires the Chaperone Network to Promote Proteostasis Upon Perturbations to the Coenzyme A and Iron–Sulfur Cluster Biosynthesis Pathways
Source: Aging Cell. 2025 Apr 30;24(6):e70038. doi: 10.1111/acel.70038 (PMC12151917; doi:10.1111/acel.70038)
Supplement: Supplementary file 1 — Data S1. [file ACEL-24-e70038-s008.pdf]

Supplement Figure Legends

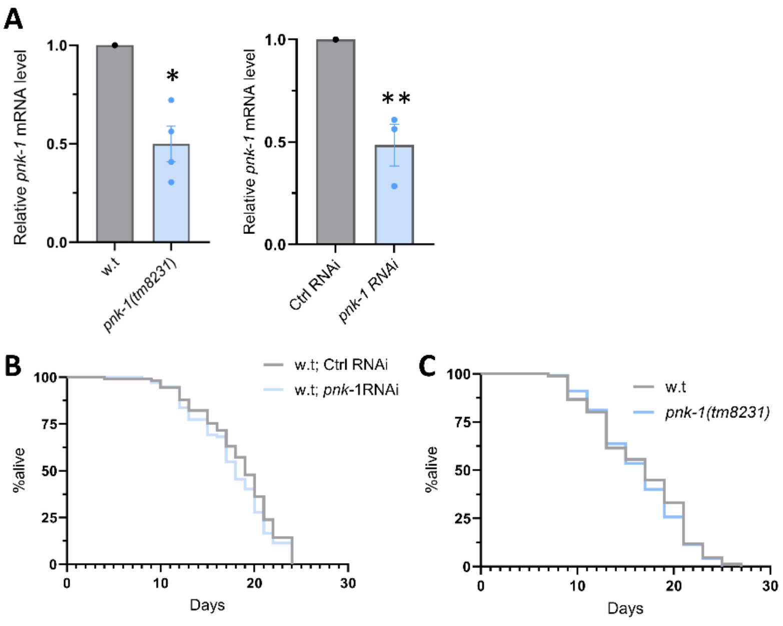

<u>Figure S1: Mild *pnk-1* deficiency does not extend lifespan of wild-type animals.</u>

(A) *tm8231* mutation is a 73 bp deletion ~3200 bp upstream of the *pnk-1* gene transcription start site. It reduces *pnk-1* transcript levels by nearly 50% as determined by qRT-PCR (N=4), as does *pnk-1* RNAi (N=3). Asterisks mark one sample t-test values. **(B-C)** Representative lifespan of *pnk-1* hypomorphic mutants. *pnk-1* RNAi treatment **(B)** and the *pnk-1(tm8231)* mutation **(C)** did not extend the lifespan of wild-type animals (N=3). Note that the data of the RNAi lifespan experiment is part of the experiment detailed in **Table S1**. Data are shown as mean ± standard error. *P<0.05. **P<0.001

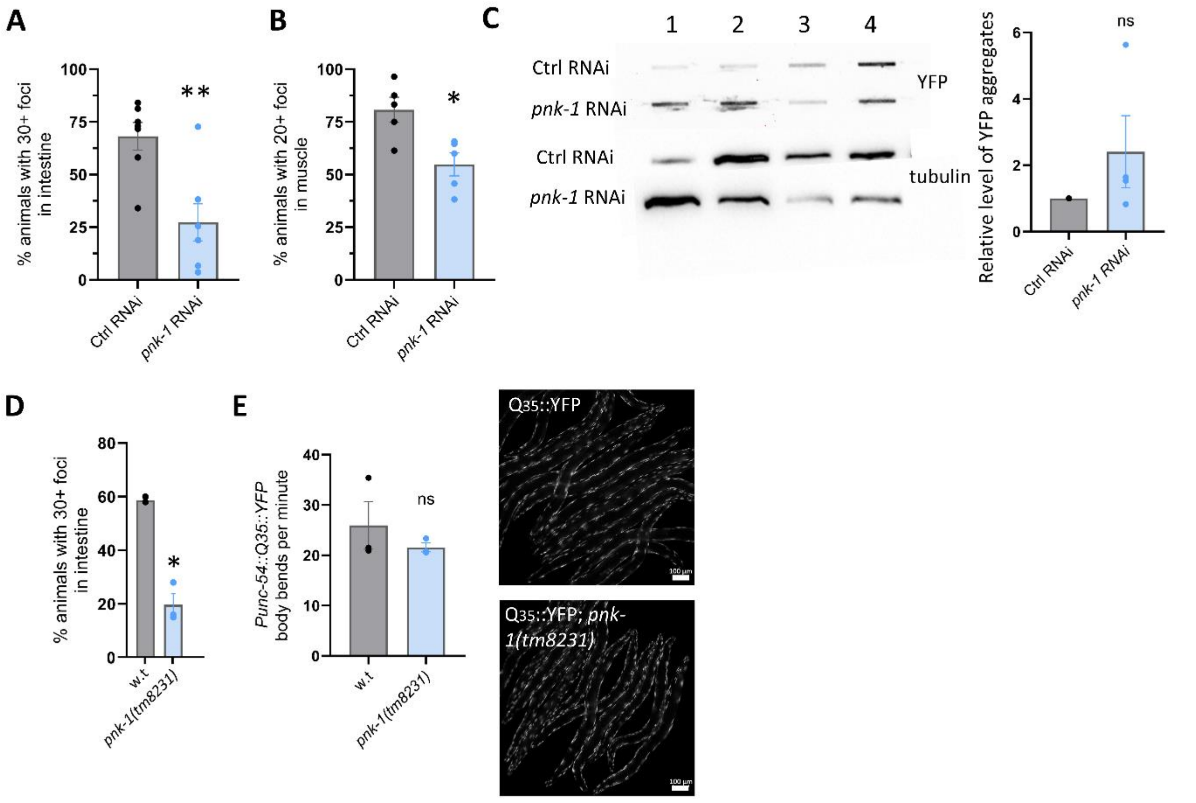

<u>Figure S2 *pnk-1* RNAi reduces PolyQ foci number without decreasing its aggregation.</u>

(A-B) *pnk-1* RNAi reduced the amount of visible foci in day 5 animals in animals expressing intestinal PolyQ$_{44}$::YFP (N=7, n>250) **(A)** or muscular PolyQ$_{35}$::YFP (N=5, n>125) **(B)**. Cochran-Mantel-Haenszel test. **(C)** Filter retardation assay of YFP aggregates from crude protein extract from PolyQ$_{35}$::YFP worms treated with control or *pnk-1* RNAi. The results shown are of four independent biological replicates. Anti tubulin western blot from the same samples is included as loading controls. one sample t-test. **(D-E)** *pnk-1* tm8231 mutation reduced the amount of visible foci in day 5 animals in animals expressing intestinal PolyQ$_{44}$::YFP (N=3, n>130), Cochran-Mantel-Haenszel test **(D),** but not in animals expressing muscular PolyQ$_{35}$::YFP (N=3, n>70), representative images of the PolyQ$_{35}$::YFP foci. Note that (D) is the control data of Figure S5C. Unpaired student's t-test.

*P<0.05, **P<0.001. Data are expressed as mean ± standard error. **P<0.001. Data are expressed as mean ± standard error.

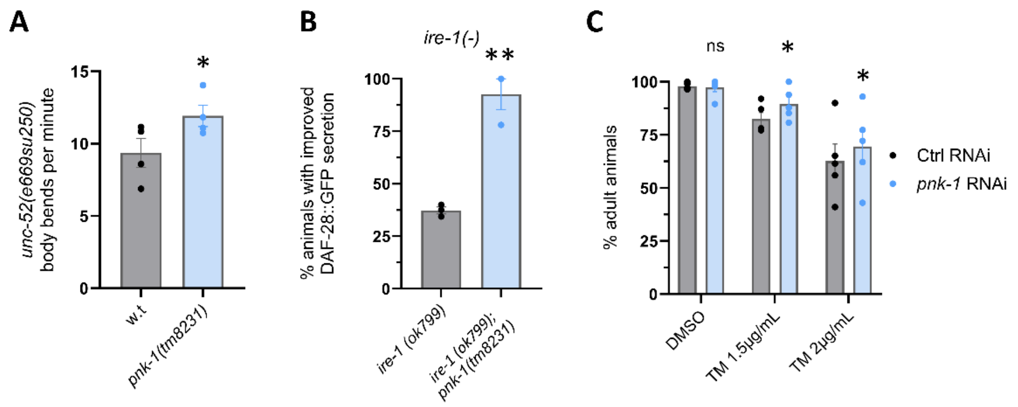

Figure S3: *pnk-1* deficiency improves proteosoatsis.

(A) *pnk-1(tm8231)* mutation improved thrashing of *unc-52(e669su250)* metastable mutants (N=4, n>100). Unpaired student's t-test. **(B)** *pnk-1(tm8231)* mutation improved the folding and secretion of a DAF-28::GFP reporter in *ire-1(ok799)* mutants (N=3, n>80). Cochran-Mantel-Haenszel test. Note that in two of the repeats, the *ire-1; pnk-1(tm8231)* strain displayed 100% animals with improved DAF-28::GFP secretion. **(C)** *pnk-1* RNAi improved animal development in the presence of the ER stress inducer tunicamycin (N=5, n>500). Cochran-Mantel-Haenszel test. Data are expressed as mean ± standard error. *P<0.05. **P<0.001.

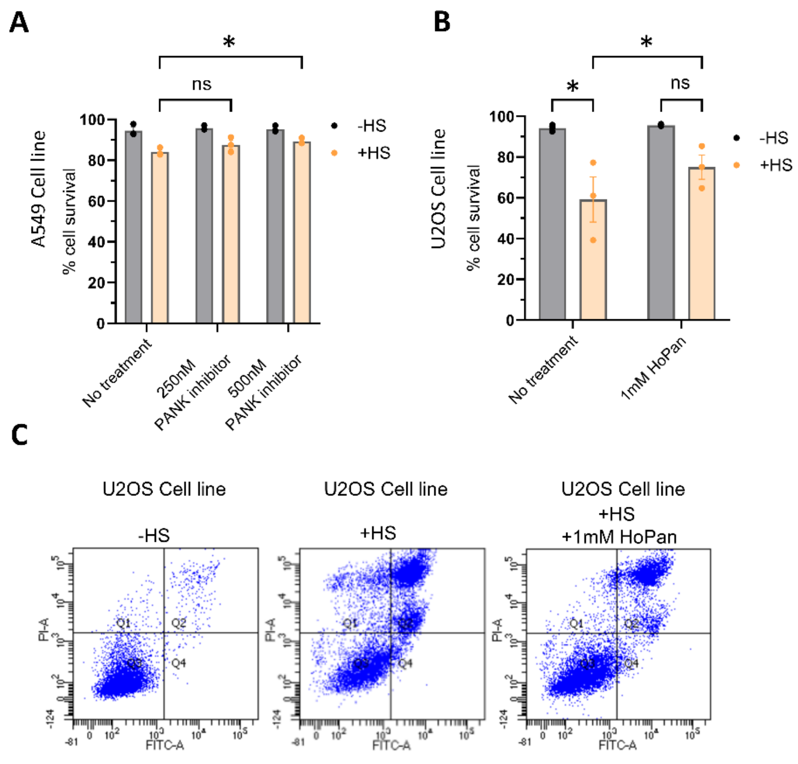

Figure S4: Inhibition of enzymes in the CoA biosynthetic pathway promotes limits heat shock sensitivity in two human cell lines.

(A) Annexin V/PI flow cytometry analysis of A549 cells following 2 hours of heat shock. Treatment with the 500nM of the PANK inhibitor increased cell resistance to heat shock (N=3). See table S2. **(B-C)** Annexin V/PI flow cytometry analysis of U2OS cells following 2 hours of heat shock. Treatment with the 1mM of the HoPan inhibitor increased cell resistance to heat shock (N=3). See table S2. Two-way Anova test. Data are expressed as mean ± standard error.

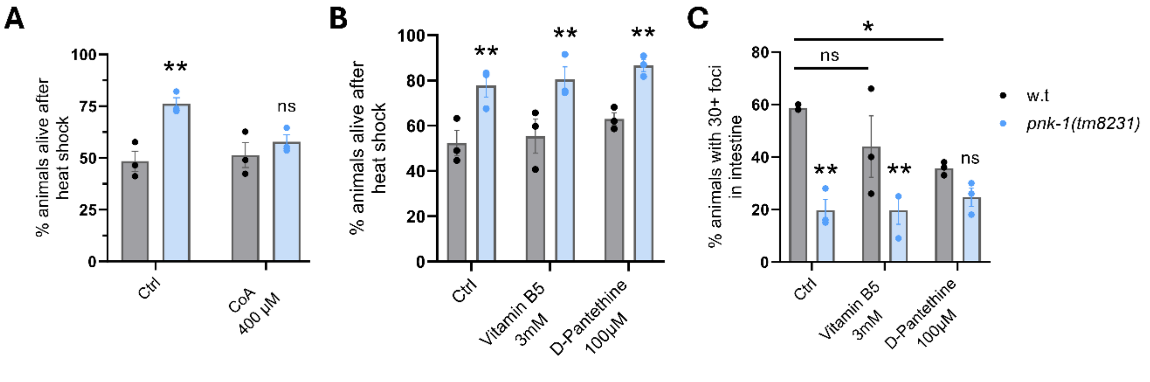

<u>Figure S5: Proteostasis improvement associated with the *pnk-1(tm9231)* deficiency is counteracted by external supplementation with CoA.</u>

(A-B) The improved heat-shock resistance of day 2 *pnk-1(tm8231)* mutants was suppressed by CoA supplementation (N=3, n>150), but not by Vitamin B5 or D-Pantethine supplementation (N=3, n>120). **(C)** The reduced foci level of day 5 *pnk-1(tm8231)* mutants was suppressed by D-Pantethine supplementation (N=3, n>120), but not by Vitamin B5 supplementation (N=3, n>120). Cochran-Mantel-Haenszel test followed by FDR correction. ns, not significant; *P<0.05. **P<0.001. Data are expressed as mean ± standard error.

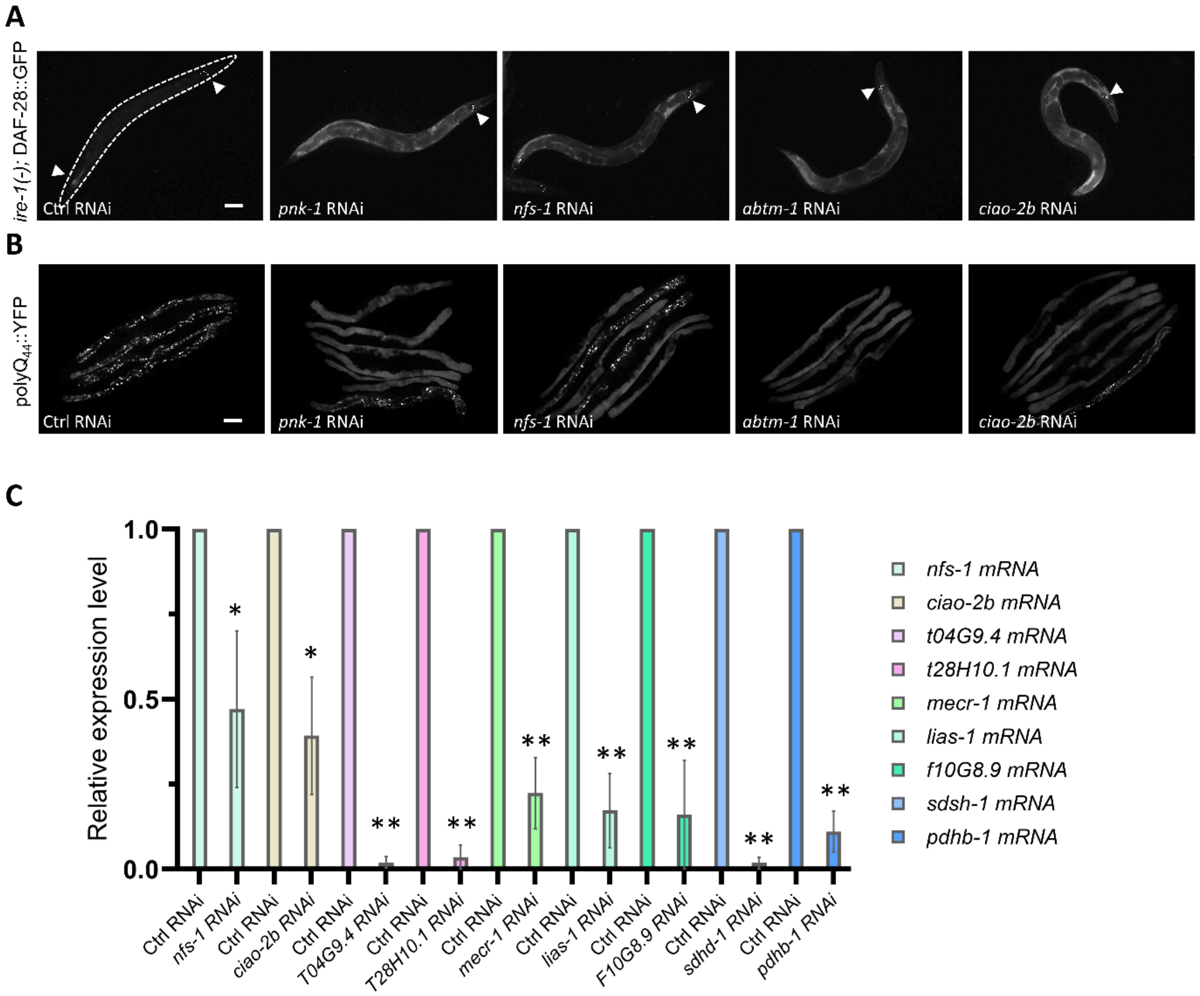

Figure S6: *pnk-1* and ISC related genes RNAi improve protein secretion and reduce intestinal PolyQ foci.

(A) Representative images of day 3 DAF-28::GFP in *ire-1(ok799)* mutants treated with *pnk-1* and ISC related genes RNAi. Arrow heads mark head neurons and intestinal cells that accumulate DAF-28::GFP protein. Knockdown of *pnk-1*, ISC (*nfs-1*) and CIA (*abtm-1, ciao-2b*) related genes improved the secretion of DAF-28::GFP. Scale bar: 100μm. **(B)** Representative images of intestinal PolyQ$_{44}$::YFP foci in day 5 adult animals treated with *pnk-1* and ISC related genes RNAi. Scale bar: 100μm. **(C)** Significant reduction in the transcript levels of the indicated genes in response to their corresponding RNAi as determined by qRT-PCR (N=3). Data are shown as mean ± standard error. Asterisks mark one sample t-test values. *P<0.05;

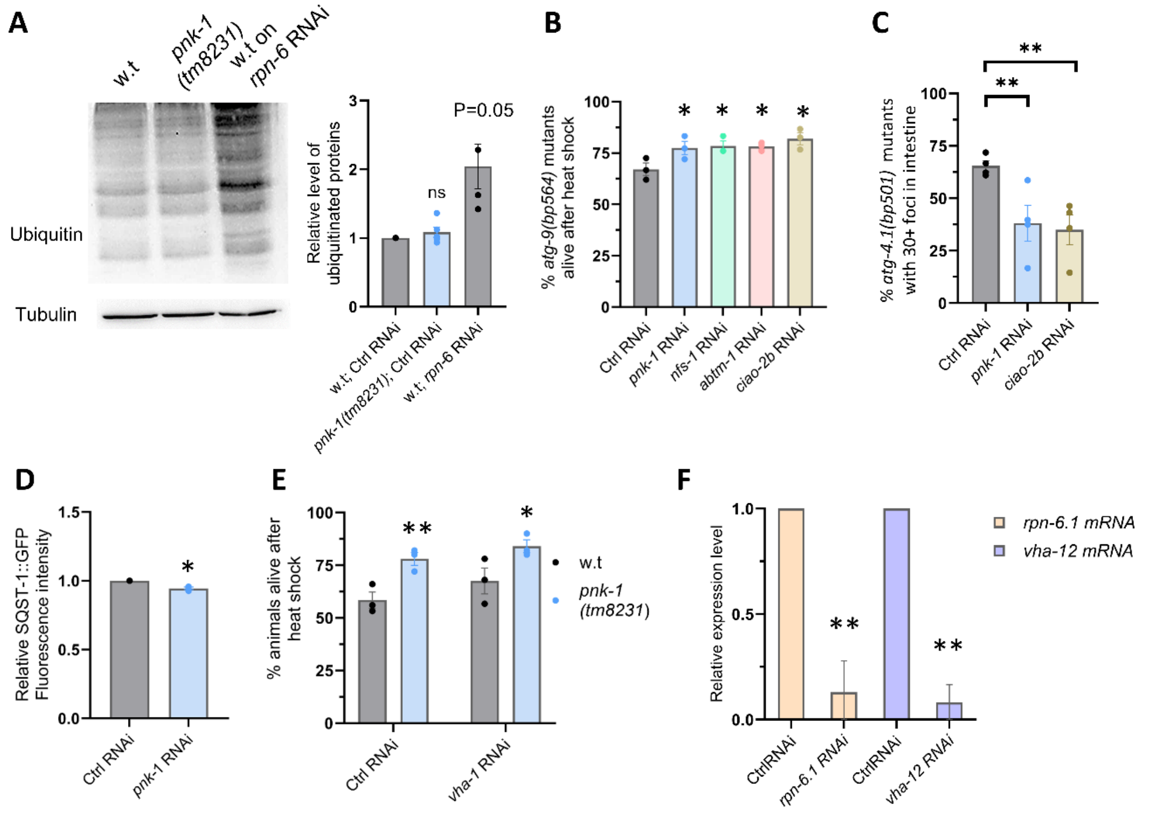

Figure S7: *pnk-1* and ISC related genes inhibition promote proteostasis independent of the proteasome and autophagy-lysosome system.

(A) The level of accumulated ubiquitinated proteins did not change upon *pnk-1* RNAi treatment. (N=4**).** One sample t-test. **(B-C)** RNAi inhibition of *pnk-1* and ISC related genes improved heat-shock resistance in *atg-9(bp564)* mutants. (N=3, n>90) (B) and reduced PolyQ$_{44}$ foci levels in the intestine in *atg-4.1(bp501)* mutants. (N=4, n>170) **(C)**. **(D)** *pnk-1* RNAi resulted in a very minor decrease in the level of the autophagy substrate reporter SQST-1::GFP. (N=3, n>90). **(E)** Inhibition of lysosome function by *vha-1* RNAi treatment did not affect heat-shock resistance of *pnk-1(tm8231)* animals. (N=3, n>90). **(F)** Significant reduction in the transcript levels of the indicated genes in response to their corresponding RNAi as determined by qRT-PCR (N=4). Asterisks mark one sample t-test values.

Data are shown as mean ± standard error. N represents the number of biological repeats, n represents the number of animals analyzed per treatment or genetic background. *P<0.05; **P<0.001. Statistical tests: one sample t-test **(A,D,F)**; Cochran-Mantel-Haenszel test followed by FDR correctin **(B,C,E)**. Comparisons were between w.t and *pnk-1(tm8231)* mutants per treatment **(E)** or relative to the corresponding control RNAi sample **(A-D)**.

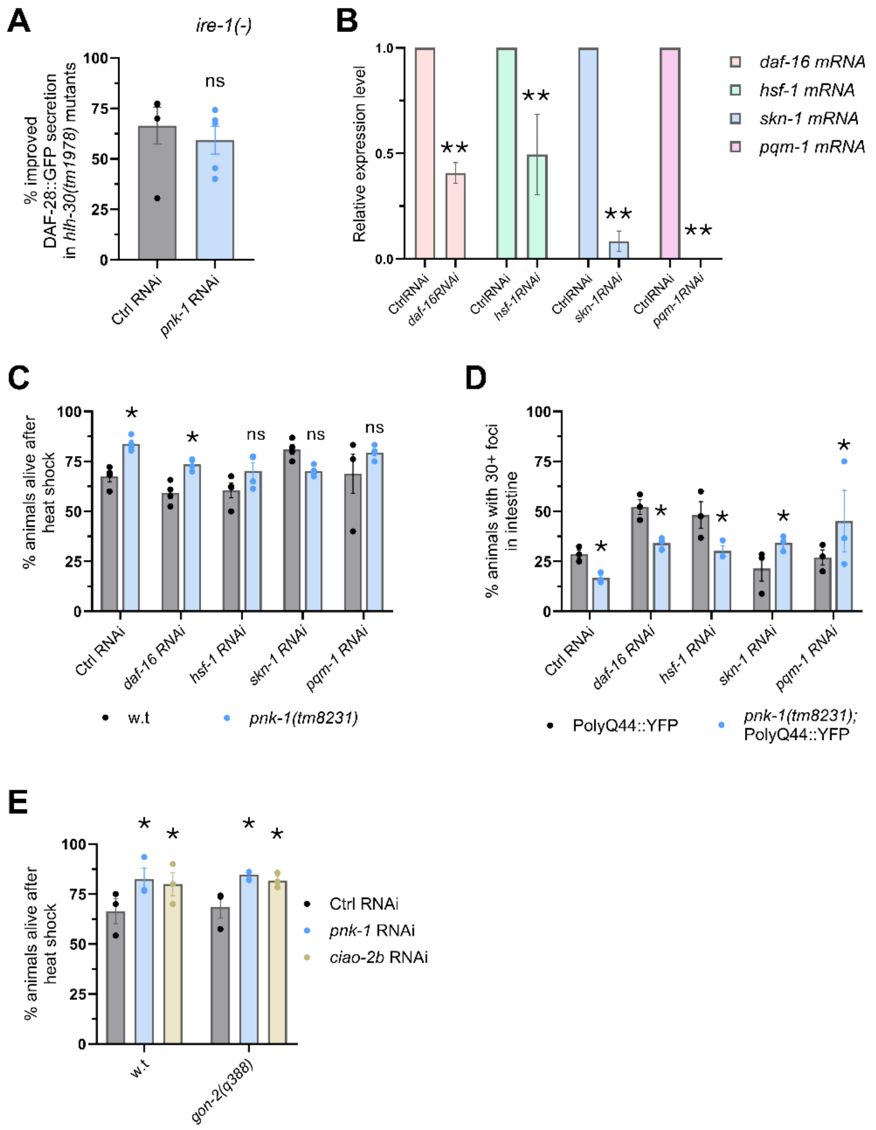

<u>**Figure S8: Stress-related transcription factors are required for proteostasis improvement upon *pnk-1* mild deficiency .**</u>

(A)*hlh-30(tm1978)* mutation prevented the benefits of *pnk-1* RNAi in the DAF-28::GFP secretion test. (N=5, n>110). **(B)** Significant reduction in the transcript levels of the indicated genes in response to their corresponding RNAi as determined by qRT-PCR (N=4). Asterisks mark one sample t-test values. **(C-D)** Stress-related transcription factors are partially required for proteostasis improvement in *pnk-1(tm8231)* mutants. (N=3-4, n>100). **(E)** *pnk-1* and *ciao-2b* RNAi treatments improve the heat shock resistance of *gon-2(q388)* gonadless animals (N=3, n>90), as it does in w.t animals. Comparisons were relative to the corresponding control RNAi sample.

(A-D) Asterisk marks Cochran-Mantel-Haenszel test values unless indicated otherwise. Data are shown as mean ± standard error.
N represents the number of biological repeats, n represents the number of animals analyzed per RNAi treatment. *P<0.05; **P<0.001.

Supplementary Tables

Table S1: The effect of *pnk-1*/ISCs partial deficiency on lifespan in *C. elegans.*
Table S2: The effect of inhibition of enzymes of the CoA biosynthesis pathway on heat shock resistance of human cell lines.
Table S3: Chaperone RNAi candidate screen.
Table S4: Data sets of potential HLH-30 CHIP-seq defined targets and chaperone and co-chaperone genes.
Table S5: Gene set enrichment analysis of the potential HLH-30 CHIP-seq targets.
Table S6: Cytosolic chaperones qRT-PCR data.
Table S7: RNAi clone data.
Table S8: qRT-PCR primers.
Table S9: Statistics.
